# The development of a recovery coaching training curriculum to facilitate linkage to and increase retention on medications for opioid use disorder

**DOI:** 10.3389/fpubh.2024.1334850

**Published:** 2024-02-15

**Authors:** Trevor Moffitt, Amanda Fallin-Bennett, Laura Fanucchi, Sharon L. Walsh, Christopher Cook, Devin Oller, Anna Ross, Molly Gallivan, John Lauckner, Jeremy Byard, Phoebe Wheeler-Crum, Michelle R. Lofwall

**Affiliations:** ^1^University of Kentucky, Substance Use Priority Research Area, Lexington, KY, United States; ^2^College of Nursing, University of Kentucky, Lexington, KY, United States; ^3^Voices of Hope, Lexington, KY, United States; ^4^College of Medicine, Center on Drug and Alcohol Research, University of Kentucky, Lexington, KY, United States; ^5^Department of Behavioral Science, College of Medicine, University of Kentucky, Lexington, KY, United States; ^6^College of Medicine, University of Kentucky, Lexington, KY, United States; ^7^Arthur Street Hotel, Louisville, KY, United States

**Keywords:** medication for opioid use disorder, peer support, opioid use disorder treatment, recovery coach, peer recovery, training

## Abstract

**Introduction:**

Medication treatment for opioid use disorder (MOUD) decreases opioid overdose risk and is the standard of care for persons with opioid use disorder (OUD). Recovery coach (RC)-led programs and associated training curriculums to improve outcomes around MOUD are limited. We describe our comprehensive training curriculum including instruction and pedagogy for novel RC-led MOUD linkage and retention programs and report on its feasibility.

**Methods–pedagogy and training development:**

The Kentucky HEALing (Helping to End Addiction Long-term^SM^) Communities Study (HCS) created the Linkage and Retention RC Programs with a local recovery community organization, Voices of Hope-Lexington. RCs worked to reduce participant barriers to entering or continuing MOUD, destigmatize and educate on MOUD and harm reduction (e.g., safe injection practices), increase recovery capital, and provide opioid overdose education with naloxone distribution (OEND). An extensive hybrid (in-person and online, both synchronous and asynchronous), inclusive learning-focused curriculum to support the programs (e.g., motivational interviewing sessions, role plays, MOUD competency assessment, etc.,) was created to ensure RCs developed the necessary skills and could demonstrate competency before deployment in the field. The curriculum, pedagogy, learning environment, and numbers of RCs trained and community venues receiving a trained RC are reported, along with interviews from three RCs about the training program experience.

**Results:**

The curriculum provides approximately 150 h of training to RCs. From December 2020 to February 2023, 93 RCs and 16 supervisors completed the training program; two were unable to pass a final competency check. RCs were deployed at 45 agencies in eight Kentucky HCS counties. Most agencies (72%) sustained RC services after the study period ended through other funding sources. RCs interviewed reported that the training helped them better explain and dispel myths around MOUD.

**Conclusion:**

Our novel training and MOUD programs met a current unmet need for the RC workforce and for community agencies. We were able to train and deploy RCs successfully in these new programs aimed at saving lives through improving MOUD linkage and retention. This paper addresses a need to enhance the training requirements around MOUD for peer support specialists.

## 1 Introduction

### 1.1 Opioid use disorder and medication for opioid use disorder

The opioid epidemic is a public health crisis with over 100,000 opioid-related overdose deaths in the United States in 2021, representing a 59% increase from 2019 (1). Kentucky had the fourth-highest state overdose death rate in 2021 (55.6 deaths per 100,000) (2). Improving access to and retention on Food and Drug Administration-approved medication treatments for opioid use disorder (MOUD), specifically methadone and buprenorphine, is critical because they decrease the risk of overdose and all-cause mortality (3). Further, treatment retention is critical because opioid use disorder (OUD) is a chronic relapsing disorder.

Despite the effectiveness of MOUD, they remain underutilized (4) often due to lack of access, misinformation, and stigma. Transportation is also a major barrier, particularly for methadone among individuals living in rural and small urban communities where there are fewer programs and longer drive times (5). Further, systemic barriers to MOUD remain pervasive in many areas of health care and the criminal legal system (6). For example, the criminal legal system has not routinely allowed persons with OUD to continue MOUD upon incarceration, a violation of the Americans with Disabilities Act (7).

### 1.2 Recovery coaching and OUD

Interventions are needed to reduce barriers to MOUD, including improving community health literacy and addressing misinformation and stigmatizing beliefs about MOUD. People who use drugs may prefer to work with peer workers (i.e., people with lived experience) versus non-peer workers (8). Individuals who choose to initiate methadone or buprenorphine often learn about them from others with OUD and report becoming interested due to their success (9). The association between shared positive lived experience on MOUD and treatment uptake, as well as frequent need for assistance navigating structural barriers to MOUD, highlight the need for a formalized recovery coach workforce with training programs emphasizing linking to MOUD and facilitating retention.

Recovery Coaches (RC; a type of peer worker) are individuals with lived experience with substance use disorder who are in remission and recovery and whose job entails performing non-clinical recovery support services, such as facilitating goal setting with participants, making resource referrals, and inspiring hope that remission and recovery are possible (10). Evidence suggests (11) RC programs can improve outcomes such as decreasing substance use (12) and increasing employment (13).

The evidence for RC-led interventions tailored to individuals with OUD, however, is limited. A randomized controlled trial of individuals (*n* = 80) treated for opioid overdose found that participants receiving RC phone support were significantly less likely to report another opioid overdose compared to participants receiving usual care (i.e., overdose education and naloxone distribution; OEND) (14). RCs also show promise in facilitating screening for illicit opioid use and interest in linkage to buprenorphine within the emergency department (15). A recent review of peer-led services for individuals with OUD identified 12 interventions, with nearly all focused on linkage to treatment (16). No studies focused on MOUD retention or RC training programs for peers working specifically with persons with OUD (16), though recent focus groups with opioid treatment program patients and staff, including RCs, demonstrated acceptability of using RCs to improve methadone retention (17). Current RC training is often limited to participation in statewide peer support certification programs and broadly described “periodic trainings” on topics like motivational interviewing (MI) and boundaries (18)–with largely absent descriptions of curriculum, instruction, and pedagogy.

### 1.3 Study overview and recovery community organizations

The HEALing (Helping to End Addiction Long-term^SM^) Communities Study (HCS) is a four-state (Kentucky, Massachusetts, New York, Ohio) parallel group cluster randomized controlled trial aiming to reduce opioid overdose deaths by 40% (19). The HCS intervention, Communities that Heal (CTH), seeks to implement evidence-based practices (EBPs) to reduce opioid overdose through community engaged, coalition-led efforts in each study community. EBPs focus on OEND, effective delivery of MOUD (with emphasis on linkage to and retention on buprenorphine and methadone), and safer opioid prescribing and dispensing (20). Eight Kentucky counties were randomized to receive the CTH intervention (January 2020 to June 2022) and eight counties were randomized to a waitlist control period, and later received the CTH intervention (July 2022 to December 2023) (19).

The HCS-Kentucky (HCS-KY) research team, recognizing the growing evidence around peer support to engage persons in treatment to promote remission and recovery, along with community interest in peer services, searched for existing training relevant to MOUD linkage and retention. After an extensive literature review and contacting national and state stakeholder groups, it was determined there was a dearth of training curriculums and resources tailored specifically to RCs around MOUD. Additionally, as only a small minority of individuals with OUD ever receive MOUD (3), many individuals entering the peer workforce do not have personal experience with or education on MOUD. To address these gaps, the KY team created two novel peer-led programs, one for linkage and one for retention, with associated comprehensive training curriculum and instruction.

The programs’ workforce was built utilizing current Kentucky state-certified Peer Support Specialists (PSSs) hired by a local recovery community organization, Voices of Hope-Lexington (VOH). Recovery community organizations are non-profit organizations that provide a breadth of recovery services, such as peer recovery support, harm reduction education, and mutual aid meetings (21). Recovery community organizations are typically independent agencies with common core values including valuing all pathways of recovery (22), allowing recovery community organizations to engage individuals in active use and across all stages of treatment readiness, remission, and recovery (23). For RC positions, VOH hired individuals who were eligible to complete the Kentucky Adult Peer Support Specialist certification (i.e., self-report being in recovery from a substance use disorder and having a GED or higher level of education). After completing the training program described below, RCs were deployed in the field as linkage and/or retention RCs as part of the EBPs chosen by the HCS-KY coalitions (24).

The purpose of this paper is to describe: (1) the development of a training curriculum for the HCS/Voices of Hope (HCS-VOH) Linkage and Retention RC Programs; (2) the HCS-VOH training curriculum contents and structure including pedagogical framework, core competencies, and learning environment; and (3) three RC case studies reporting about their training experiences along with the number of RCs trained and deployed.

## 2 Methods–program overview, pedagogical framework and principles, underlying competencies, and trainee experience collection

### 2.1 Overview of HCS-VOH linkage and retention RC programs

The overarching goals of the HCS-VOH Linkage and Retention RC Programs (see Table 1) are to reduce opioid-involved overdose deaths by helping participants enter and remain in MOUD treatment, respectively, with active assistance in addressing barriers to these goals. In both programs, RCs aim to build health literacy around OUD and MOUD, destigmatize MOUD, increase recovery capital, and set recovery goals alongside providing OEND and harm reduction education. Linkage RCs are deployed in community settings to enroll individuals at high risk for opioid overdose (e.g., syringe service programs, criminal legal system venues such as detention centers, etc.). During initial visits, RCs educate participants about Food and Drug Administration-approved medications for OUD and address common misconceptions about being on MOUD. RCs attempt ongoing daily contact with participants until they successfully attend a first appointment with a MOUD provider, after which point the RC attempts weekly contact.

**Table 1:** Description of HEALing Communities Study (HCS)–Voices of Hope (VOH) linkage and retention recovery coaching (RC) program.

Description	Linkage program	Retention program
*Goal*	Reduce opioid-involved overdose deaths by helping participants connect to medication for opioid use disorder (MOUD) treatment	Reduce opioid-involved overdose deaths by helping participants remain in MOUD treatment
	Provide overdose education and naloxone distribution Build health literacy around opioid use disorder/MOUD Provide harm reduction education Increase recovery capital Destigmatize MOUD Set recovery goals	
*Frequency of Contact between Participant and RC*	After intake, prior to attending first appointment with MOUD provider – **daily** tele-contact attempts After attending first appointment with MOUD provider – **weekly** tele-contact attempts for 30 days, then linkage program discharge with option to transfer to retention program if available	After intake, during the first 30 days – **weekly** tele-contact attempts After 30 days in the program, contact is based on a risk assessment completed every 30-days
*Telephone Recovery Support*		In addition to their regular RC contacts, retention program participants receive **weekly** telephone recovery support from RCs. These calls are open-ended conversations initiated with, “How is your recovery going today?”
*HCS-funded Resources for RC Use*	Housing assistance Up to two weeks of temporary/emergency housing One-time rent assistance Transportation assistance for MOUD appointments or other recovery-related destinations Fuel cards Bus passes Direct transportation from VOH peer drivers (i.e., drivers who are in recovery) Miscellaneous barrier relief funds approved on *ad hoc* basis by HCS to facilitate treatment for participants who do not qualify for other community support programs (e.g., government ID fees, utilities, medical visit, prescription co-pay)	
*Discharge*	Participants are discharged after 30 days of MOUD treatment and may be enrolled into the retention program. A warm-handoff to a retention RC is provided when possible.	Participants can remain in the retention program for as long as desired.

Retention program RCs focus on increasing recovery capital and addressing barriers and concerns that may adversely affect participants’ retention in MOUD treatment and are predominantly embedded in community MOUD provider agencies, though some are also deployed in probation and parole programs to help retain those who are already on MOUD. During the initial intake, RCs focus on understanding participant concerns and barriers to staying in MOUD treatment and complete a recovery capital assessment to learn about participants’ potential supports and recovery goals. RCs contact participants at least weekly during the first 30 days to address retention barriers such as transportation, housing, employment, insurance, obtaining government identification, etc. Subsequently, RCs complete a risk assessment every 30 days to inform the recommended frequency of RC-participant contact based upon potential risk for treatment discontinuation (Table 2). Regardless of risk level, participants receive weekly RC telephone recovery support calls designed to provide connection to resources, non-judgmental social support, and growth of the recovery support network (25). RCs rotate making telephone recovery support calls and contact all enrolled participants. RCs in both the Linkage and Retention programs can also access the study’s barrier relief fund to assist with participants’ barriers to starting or continuing MOUD. These miscellaneous requests are approved by HCS on an *ad hoc* basis to facilitate treatment, such as providing a phone or paying for a government ID, utilities, medical visit, or prescription co-pays.

**Table 2:** Risk assessment for retention program participants.

Level 1 Low risk	Participant is stable, meaning **ALL three self-report criteria are met:** Missed zero medication for opioid use disorder (MOUD) appointments in last 30 days, Reports no concerns regarding their MOUD, AND Reports no current barriers to MOUD treatment retention Participant is followed telephonically **monthly and as needed** as long as participant desires or no longer meets low risk criteria
Level 2 Moderate risk	Participant **meets ANY of these three self-report criteria:** Missed **one** MOUD appointment in last 30 days, Reports some concerns about their MOUD, **OR** Reports **some barriers** to MOUD treatment retention Participant is followed telephonically or in-person **every two weeks and as needed** as long as participant desires or no longer meets criteria
Level 3 High risk	Participant **meets ANY of these three self-report criteria:** Misses **multiple** MOUD appointments within last 30 days, Continues to express difficulty with their MOUD, **OR** Reports **numerous barriers** to staying in MOUD treatment Participant is followed telephonically or in-person **weekly and as needed** as long as participant desires or risk level improvement

The HCS protocol (Pro00038088), which includes the HCS-VOH Linkage and Retention RC Programs, was approved by Advarra Inc., the HEALing Communities Study single Institutional Review Board (IRB). Written informed consent for all individuals receiving HCS services, (e.g., linkage and retention program participants) was waived by the IRB.

### 2.2 Training curriculum development

After an extensive literature review and contacting key stakeholder groups including the SAMHSA-funded Providers Clinical Support System (26) and Opioid Response Network, and Kentucky’s Department for Behavioral Health, Developmental and Intellectual Disabilities, it was determined there was a dearth of training resources tailored specifically to RCs around MOUD. As a result, the HCS-KY team, including physicians who are board-certified in addiction medicine, and Voices of Hope iteratively built the training curriculum beginning with an initial version used at the start of the study’s intervention period (January 2021). As the linkage and retention programs grew and more RCs were hired, the group, whose members had expertise in MOUD treatment, recovery support services, implementation science, and the criminal legal system, worked collaboratively to assess the training curriculum’s needs and adjusted accordingly. Weekly meetings were held with HCS and VOH leadership in which cases and/or issues from the field were discussed. Based on these discussions and the training curriculum’s learning objectives, changes and additions were made to the curriculum and the programs’ standard operating procedures. The final version of the training curriculum is ~150 h (Table 3) though some placements require extra training. For instance, RCs deployed to detention centers or specialty courts have additional training specific to these venues. The detailed training manual is provided in the Supplementary Material (Data Sheet 1) and available online.^1^

**Table 3:** Training for HCS-VOH linkage and retention recovery coaching programs.

Training name	Created by	Description	Length/Self-guided or In-person	Domain/Category
VOH standard trainings				
Kentucky (KY) adult peer support specialist certification	KY Cabinet for Health and Family Services	KY initial and continuing Adult Peer Support Specialist certification training requirements (908 KAR 2:220). Certification is often transferrable to other agencies for future RC positions.	30 h/Live, Online or In-person initial; 6 h/year continuing education	PRSCC^1-12^
Voices of Hope (VOH) orientation	VOH	Introduces RCs to the culture and history of VOH, general guidelines and core values, and employee policies and procedures. This provides a sense of community and purpose from day 1.	6 h/Live, In-person	PRSCC^1-3^
Recovery coach academy (RCA)	Connecticut Community for Addiction Recovery (CCAR)	Discusses stages of change and stages of recovery, dimensions of recovery and recovery coaching, and recovery wellness planning.	30 h/Live, In-person	PRSCC^1-12^
Question, persuade, refer (QPR) suicide prevention training	QPR Institute developed by National Alliance on Mental Illness	Dispels myths about suicide, teaches how to understand and recognize suicide warning signs, and responses to someone who is potentially suicidal.	1 h/Live, Online or In-person	MH, PRSCC^4-6^
Basic life support training	American Heart Association	Teaches how to respond to a cardiac arrest, respiratory distress, or an obstructed airway.	4 h/Live, In-person; 2 h/Self-paced, Online	ER
Internal emergency protocol training	VOH	Trains RCs on scenarios including medical emergencies, active violence, suspicious persons, and inclement weather, including steps to follow to protect the health and safety of staff, guests, and physical property.	0.5 h/Live, In-person	ER
Human subjects protection (HSP) training	CITI program	Explains ethical practices to ensure HSP and well-being, history, and importance of ethical conduct in research. Research involvement is new for many RCs.	4 h/Self-paced, Online	RSA
Self-management and recovery training (SMART) recovery and SMART family and friends facilitation training	SMART	Includes 4-Point Program, various pathways to recovery, SMART tools, and meeting facilitation. RCs shadow a SMART meeting prior to facilitating. SMART supports all recovery pathways including medication for opioid use disorder (MOUD).	40 h/Self-paced, Online	PRSCC^7-8^
SMART recovery meeting protocol overview	SMART	Provides guidance on implementation of SMART meetings. Upon completion, the RC can facilitate a SMART recovery meeting, record SMART recovery meeting information for study reporting, and identify potential community partners/champions for sustainability.	0.5 h/Live, Online or In-person	RSA, PRSCC^6-8^
Information and support line protocol overview	VOH	Trains RCs to answer and properly document all support line calls. This non-crisis line operates during standard business hours. RCs learn to differentiate between crisis and non-crisis calls and to utilize motivational interviewing (MI) skills to offer support.	1 h/Live, Online or In-person	MH, ER, PRSCC^4-6,8^
Telephone recovery support (TRS) didactic training and shadow shift	VOH	RCs shadow an experienced and trained RC in TRS, conduct calls under supervision, and then make independent TRS calls.	2 h/Live, In-person	PRSCC^4-12^
Harm reduction recovery training	VOH and materials from the National Harm Reduction Coalition, including Getting Off Right*	RCs learn about harm reduction as a recovery pathway, discuss practical harm reduction strategies and importance of changing language to address stigma surrounding people who use drugs.	4 h/Live, In-person	OUD, SUD, PRSCC^3,7,8,11^
The Substance Abuse and Mental Health Service Administration (SAMHSA) provider clinical support system training				
Substance use disorder 101 training**	The providers clinical support system	These modules provide a broad look at the evidence-based practices around substance use disorder treatment, prevention, and screening.	1 h per module/Self-paced, Online	SUD, OUD, PRSCC^3,4,8^
Materials created for HCS-VOH program				
Medication for opioid use disorder: What clients ask RCs	HCS treatment team	Explains MOUD to RCs, including some common participant questions about MOUD and possible responses.	0.5 h/Self-paced, Online	OUD, PRSCC^3,4,6,8,11^
OUD Regulatory Training for RCs	HCS treatment team	Reviews MOUD treatment regulations and confidentiality laws so RCs can explain this to participants (e.g., methadone treatment visit frequency). Curriculum covers informed consent, HIPAA, 42 CFR Part 2, the Drug Enforcement Administration, and KY regulations for opioid treatment programs and office based opioid treatment pertinent to linking to and helping clients stay retained in MOUD.	0.5 h/Self-paced, Online	OUD, PRSCC^3^
Motivational interviewing workshop	HCS treatment team	RCs practice MI techniques to help increase participant engagement.	2 h/Live, Online or In-person	PRSCC^3,6,7,9,11,12^
Training on standard operating procedures for data collection	VOH-HCS	RCs review data collection standard operating procedures and discuss sample cases and practice data entry.	4 h/Live, In-person	RSA, PRSCC^3^
Community resource guide orientation	HCS treatment team	Review use of community resource guides with clients. Community resource guides contain local resources such as social support services; food assistance programs; housing; and MOUD, OEND, and syringe service programs.	0.5 h/Self-paced, Online	PRSCC^3,4^
Overdose education and naloxone distribution (OEND) training	HCS implementation science	This training describes the HCS OEND program and the data reporting requirements for HCS.	0.5 h/Live, Online	RSA, PRSCC^3,4^
Release of information training	HCS criminal legal system (CLS) team	The voluntary HCS release of information allows client information to be shared among HCS RCs to help continue care with potential placement changes (e.g., incarceration) and/or transitions from linkage to retention programs.	0.5 h/Self-paced, Online	RSA, PRSCC^3^
Opioid treatment program (OTP) tour (*for RCs deployed in OTP settings*)	HCS treatment team	To understand the day-to-day operations of OTP, RCs tour a local OTP with HCS staff and learn about workflow, rules, and staff roles.	1 h/Live, In-person	RSA, PRSCC^1,3,4^
Criminal Legal Venues Involved in HCS (*for RCs deployed in criminal legal system settings*)	HCS criminal legal system team	Reviews the relationship between OUD and the CLS, acquaints RCs to relevant CLS venues, and provides context for education and working with participants involved in the CLS	1 h/Self-paced, Online	RSA, PRSCC^1-4^
Overview of court processes and custodial changes (*for RCs deployed in CLS settings*)	HCS CLS Team	Reviews when and how participants may experience a pre- and post-sentencing change in custody during the adjudication process. Explains key court hearings impacting release dates in preparation for linkage upon release. Explains importance of coordination and communication between participants and their public defender/alternate sentencing workers pre-release or parole officers post-release about their treatment desires.	0.5 h/Self-paced, Online	RSA and PRSCC^3,4^
MOUD competency	VOH-HCS	Part 1 – Orientation video to the HCS MOUD Flyer and how it should be used with participants narrated by HCS physician and sample role play video using MOUD flyer Part 2 – Two, 1-h training sessions with an experienced RC building on the orientation videos in Part 1. The first session reviews the HCS MOUD Flyer, and the second session introduces the role play scenarios used in the final competency checklist. Part 3 – Skills-building workshop with a person with lived experience who has supervised teams of peer support specialists and is a MI Network of Trainers certified trainer. Topics include: active listening, appropriate disclosure of personal stories, addressing common questions around MOUD, harm reduction techniques, and guidance for active linkage using the HCS MOUD flyer. Part 4 – Interactive role plays with trainees and trainers incorporating skills and content from Part 3 including addressing common questions about MOUD and using MI techniques Part 5 – Prep sessions for the final competency checklist led by an experienced RC. Sessions review the MOUD Flyer content, role play scenarios, and the competency rubric Part 6 – Competency check assessing if RC can clearly explain the MOUD flyer and facilitate a linkage to MOUD and the main components/contact frequency of both linkage and retention programs	0.75 h/Self-paced, Online ~2 h/Live, In-person or Online 8 h/Live, In-person 8 h/Live, In-person ~2–4 h/Live, In-person or Online 0.75 h/Live, Online	OUD, PRSCC^3,4,8,9,11^
RC boundaries training	VOH-HCS	This training educates RCs on important concepts related to professional boundaries. RCs are given case studies with real-world boundary crossing scenarios to practice navigating along with a framework for resolutions.	1.5 h/Live, In-person	PRSCC^1,3,5–9^

ER, Emergency response; MH, Mental health; OUD, Opioid use disorder; RSA, Research and study activities; SUD, Substance use disorder. PRSCC, Peer recovery support core competency categories (32): ^1^Support collaboration and teamwork, ^2^Promote leadership and advocacy, ^3^Promote growth and development, ^4^Link to resources, services, supports, ^5^Help peers manage crises, ^6^Value communication, ^7^Provide support, ^8^Provide information about skills related to health, wellness, and recovery, ^9^Engage peers in collaborative, caring relationships, ^10^Share lived experiences of recovery, ^11^Personalizes peer support, ^12^Supports recovery planning. *National Harm Reduction Coalition (https://harmreduction.org/) and Getting Off Right (https://harmreduction.org/issues/safer-drug-use/injection-safety-manual/). **Specific modules included are: changing language to change care: Stigma and substance use disorders, overview of substance use disorders, medication for opioid use disorder, introduction to the criminal justice system and medication for opioid use disorder, preventing opioid-involved overdose with education and naloxone, principles of motivational interviewing: useful for primary care providers.

RCs begin by completing Kentucky state-certified Adult Peer Support Specialist (PSS) training. Supplementing the Kentucky PSS program was important given its brevity (30 required hours) and the cursory teachings of essential topics such as MOUD, substance use disorder, effective listening skills, and principles of recovery. Next, RCs complete VOH’s harm reduction training and an array of nationally recognized trainings, such as SMART Recovery (27) and several Provider Clinical Support System Substance Use Disorder 101 training modules (26). We also created training on topics such as MI skills, boundaries, and OUD/MOUD because RCs often have limited experience with MOUD and may hold stigmatizing views toward MOUD, and many agencies are not familiar with the RC-role and may ask RCs to perform tasks that are outside their scope of work (i.e., boundary violations). All RCs attend weekly team supervision with their program coordinators, receive additional supervision as needed, and regularly review the programs’ Standard Operating Procedures. RCs are required to participate in weekly sessions of the Kentucky Overdose Prevention Education Network, a series of HCS-created live virtual didactics covering relevant topics such as trauma and suicide prevention.

### 2.3 Pedagogical framework and principles

To build an RC workforce competent and comfortable discussing MOUD with potential and current participants, our training curriculum employs a variety of pedagogical methods. The breadth of methods provides trainees multiple opportunities to demonstrate their understanding of critical concepts (e.g., MOUD, motivational interviewing) in active learning formats. Because a major focus of the training is supporting all recovery pathways, including MOUD treatment, it was crucial that our pedagogy aligned with an inclusive teaching approach for our training. Inclusive teaching pedagogy recognizes the diverse lived experiences of learners and leans into the many beliefs individuals bring to the learning environment (28). Trainees may bring past experiences of MOUD or hold conscious or unconscious stigmatizing beliefs about MOUD (e.g., “Taking MOUD is trading one drug for another”) as they begin their training. Our inclusive approach recognized these experiences and beliefs and, rather than invalidate their lived experience, invited trainees to build skills and knowledge necessary to support others with different recovery pathways (MOUD, specifically).

The breadth of the training’s inclusive pedagogical methods is most apparent in the summative assessment, the MOUD Competency, a six-part, ~23-h training and evaluation. In Parts 1 and 2, trainees are introduced to and review with an experienced RC the HCS MOUD flyer (Supplementary Image 1), which was created by the 4-state HCS Consortium and covers terminology like remission and recovery, understanding opioids, how each of the three MOUD work, and common questions (e.g., MOUD effects on opioid withdrawal and cravings). In Part 3, trainees are introduced to motivational interviewing (MI) by a nationally certified Motivational Interviewing Network of Trainers facilitator. The facilitator collaboratively establishes ground rules with trainees, an important step in creating a positive classroom climate (29), and engages trainees in active learning through discussion and retrieval practice. Formative assessments, those that encourage active participation from trainees (30), are held through informal role plays throughout Parts 2 and 3.

Part 4 of the MOUD Competency focuses on using MI skills with in-depth role plays (~30 min each) of a coach-participant interaction in Linkage and Retention programs. In line with a transparent assignment design (31), trainers share: (1) the purpose of the role plays (i.e., transferability to real-world participant interactions), (2) the description of the role-play activity and what is expected from trainees, and (3) a rubric with criteria by which trainees are graded and required to pass before deployment. The role-play scenarios and rubric mirror the summative assessment, giving trainees a clear understanding of what will be expected. A checklist outlining required competencies (e.g., describe OUD as a chronic illness) is provided for each role play. During role plays, coaches practice MI skills, share their lived experience with discretion to inspire hope, review the MOUD flyer, and answer questions drawing on their knowledge of harm reduction and MOUDs. RCs are taught to use their MI skills to guide how they share their story for the participants’ benefit as well as respond to participant questions and concerns. The same two trainers (AFB, JB) led Parts 4 and 5 throughout the program’s duration. During Part 5, RCs are given additional practice opportunities and time to prepare for the competency check with an experienced RC.

The training’s summative assessment, Part 6, is a ~ 45-min competency check led by a RC supervisor alongside a study physician to support and clarify questions as needed. The RC supervisor asks trainees questions to demonstrate proficiency and comfort in explaining the MOUD flyer and using its information to address frequently asked questions (e.g., “Do I have to go through withdrawal to start MOUD?”). Trainees also role play one of three scenarios answering common participant questions while demonstrating their MI skills and knowledge of linkage and retention programs. The physician and RC supervisor are together responsible for passing or requiring the RC, per the grading rubric (Supplementary Image 2), to have more training before reassessing competency for field deployment. The same study physician (ML) led Part 6 throughout the program’s duration.

### 2.4 Underlying competencies

Our training curriculum aligns with the core competency categories for peer workers in behavioral health services established by the United States Substance Abuse and Mental Health Services Administration (32) (see Table 4). Kentucky’s peer support specialist certification, which all trainees are required to complete, aligns with all 12 core competencies, as does the Recovery Coach Academy developed by the Connecticut Community for Addiction Recovery (33). The rest of the trainings meet at least one core competency category or are intended to supplement typical RC training for working on a research study or in the field (e.g., human subjects protection training or basic life support training). Specific core competency categories met by each training are listed in Table 3.

**Table 4:** Underlying curriculum core competencies.

Core competency categories for peer workers in behavioral health services^1^
Engages peers in collaborative and caring relationships
Provides support
Shares lived experiences of recovery
Personalizes peer support
Supports recovery planning
Links to resources, services, and supports
Provides information about skills related to health, wellness, and recovery
Helps peers to manage crises
Values communication
Supports collaboration and teamwork
Promotes leadership and advocacy
Promotes growth and development

^1^Substance Abuse and Mental Health Services Administration (32).

### 2.5 Collecting trainee experiences

To inform curriculum modification and improvement, we interviewed three RCs referred by RC supervisors who had been placed at their sites for at least 6 months. To maintain confidentiality, one team member (TM) without a RC supervisory role reviewed the referrals, chose three RCs with diverse agency placements, including experience with linkage and retention programs, and conducted the interviews. RCs data were de-identified, summarized, and shared for their review for accuracy prior to team dissemination. Questions included general impressions of the training, how it prepared them (or not), how it changed their views on MOUD (or not), and how the MOUD flyer was used in their work (Supplementary Table 1). IRB approval to collect case studies was received prior to interviewing RCs.

## 3 Learning environment and learning objectives

### 3.1 Learning environment

The curriculum is a hybrid design with a blend of in-person, asynchronous online, and synchronous online training. In-person training emphasize active learning through interactive components like the discussion and role plays in the Recovery Coach Academy and MOUD Competency. Using asynchronous online instruction for several introductory trainings helped reduce the burden on training staff and promoted inclusivity for RCs who had different paces of learning. Online training, both asynchronous and synchronous, were also beneficial as trainees were spread across several counties in Kentucky. All trainings were completed during normal work hours (i.e., compensated time) and, for online training, on work-issued computers. Lengthier in-person training (e.g., Recovery Coach Academy and Parts 3 and 4 of the MOUD Competency), were scheduled once per month so that several RCs could attend at once. Larger groups attending these training helped to both reduce trainer burden and encourage active discussions. RC supervisors assisted trainees in scheduling required in-person training offsite (i.e., Kentucky state-certified Adult Peer Support Specialist and Basic Life Support Training).

### 3.2 Learning objectives

The training program’s learning objectives were defined and outlined for trainees in the MOUD Competency rubric (See Supplementary Image 2). By the end of the training program, trainees should be able to: (1) explain key principles from the HCS MOUD Flyer (e.g., each MOUD mechanism of action, how to access MOUD, effects of each MOUD), (2) successfully demonstrate MI skills by using open-ended questions, affirmations, reflections, and summaries while discussing MOUD, (3) provide important information on harm reduction and related resources to participants (e.g., overdose education, naloxone access, safe injection, syringe service programs), and (4) explain the linkage and retention programs (e.g., purpose of each program, frequency of contact).

## 4 Results and coach experiences

### 4.1 Descriptive quantitative results

From December 2020 to February 2023, 93 RCs and 16 RC supervisors completed the training program. Two individuals completed the MOUD Competency evaluation twice before passing, and two ultimately failed and were not able to be placed within an agency. Both individuals who failed were not able to complete the final competency assessment due to personal reasons and did not participate in re-training. RCs were deployed at 45 agencies in linkage and retention programs across the eight initial counties from December 2020 through December 2022 at varying effort levels determined based upon agency need (Table 5). Most agencies (*n* = 31, 72%) chose to sustain the programs’ RC services through a state-funded grant after December 2022. The training materials developed by HCS, including a detailed training manual (see Supplementary Material (Data Sheet 1)), have been provided to VOH for continued internal use to enhance sustainability. Currently, there are 24 agencies within the second set of communities randomized to the intervention where HCS RCs are deployed.

**Table 5:** RC deployment sites, program, and effort by agency type.

Agency type	Program	Number of agencies	Average hours per week
Behavioral health (e.g., addiction counseling services)	Linkage	2	20.0
Detention center	Linkage	4	40.0
Emergency department	Linkage	1	40.0
Intensive outpatient addiction treatment	Retention	1	40.0
Office-based opioid treatment	Retention	19	17.3
Opioid treatment program*	Retention	5	22.7
Probation and parole	Retention	1	10.0
Recovery community center	Linkage	1	5.0
Recovery housing	Retention	1	10.0
Residential treatment	Retention	1	32.0
Specialty court	Linkage	3	18.3
Shelter	Linkage	2	30.0
Syringe support program	Linkage	4	17.3
Total		45	302.6

*Federally licensed methadone providers.

### 4.2 Recovery coach training program experiences

Two RCs commented that the training program was unlike any other training experience, specifically the number of interactive portions and emphasis on skills-building. RC #2 described the interactive components (e.g., role plays in the MOUD Competency) as, “the best [training] experience at any job I’ve ever had… a lot of workplaces will throw you in, but having one-on-one [role plays to practice] was very helpful.” RC #3 explained that the MI skill building was noticeably different from trainings at previous jobs: “in other recovery jobs you are taught to be their friend and have to adapt skills on your own… this training taught me a whole other way to talk to people.”

RCs were also asked how the training program did or did not prepare them to help participants with MOUD linkage and/or retention. RC #1 commented that the MOUD Competency process was especially helpful in addressing misconceptions or stigma: “It’s stigmatized… [potential participants] say ‘you are not really sober if you are on these medications’… now I can explain the difference between opioid [physical] dependence and opioid use disorder… it’s good we go over it in detail like we do.” RC #3 reported hearing similar stigma at their linkage site (“you are trading one drug for another”) and shared that the HCS MOUD Flyer helped educate an individual who “was intrigued but wanted a better understanding” of how MOUD works. At their retention site, RC #2 was able to use their training and explain how buprenorphine works including the ceiling effect as a partial agonist to a participant who was still confused, “when she took buprenorphine why she was wasn’t getting the same effects [as the opioid she had been using].”

Finally, RCs were asked if the training challenged any previous views they had around MOUD and harm reduction. Two RCs came from a 12-step background and reported that the training program challenged their views around abstinence and MOUD. RC #2 reported that after completing the training program their “whole perspective has changed. I understand addiction better, understand chronic illness better, the disease process, how the medications work, and see how they help people live successful lives.” RC #1 explained that previously they viewed many harm reduction components as “enabling,” but was challenged during the Harm Reduction as a Recovery Pathway Training: “[the trainer] asked me ‘Well do you want [people in active opioid use] to die?’ and I said ‘No’… so it opened my eyes to look at it in a different light.”

## 5 Discussion and lessons learned

### 5.1 Discussion

We described the development of the novel training curriculum for the HCS-VOH Linkage and Retention RC Programs that provided education and skill-building for discussing MOUD with potential and enrolled participants. The training program supplemented the state-level peer support specialist certification with more in-depth education on topics such as MOUD, substance use disorder as a chronic disease, harm reduction, boundaries in peer support, and human research ethics. Specific skills-development training utilized role plays and allowed for demonstration of competencies and feedback in MI, OEND, and data collection. RCs demonstrated both their skills and knowledge in a final MOUD Competency evaluation.

The training curriculum is novel in two important ways. First, it focused on building a robust RC workforce aimed at addressing the opioid epidemic, specifically by increasing RC health literacy around MOUD and focusing on linkage to and retention on MOUD. Previous RC interventions that focused on OUD and MOUD outcomes were limited to linkage, did not address MOUD retention (16), or assure adequate RC health literacy around MOUD. Training RCs only for linkage to MOUD and not for retention leaves a critical gap in the continuum of care (18), and our retention program addresses this gap. This gap could also be addressed at the policy level by state-level stakeholders responsible for peer support specialist certification who could enhance competency requirements around MOUD.

Second, our training program addresses the dearth of comprehensive, standardized training for RCs in peer-based OUD interventions in the literature (11, 34). Training RCs to address MOUD misconceptions was especially important in addressing stigma in the community as evidenced by our case studies (“whole perspective has changed” RC#2) and emerging research on MOUD stigma (35, 36). We created components (e.g., MOUD Competency) so that all RCs would have the same, standardized training for discussing MOUD with participants, improving on previous studies that did not report extensive MOUD-specific training for their RCs (13, 37). Building this knowledge base around MOUD is critical for the RC workforce as more RC-led interventions are deployed for OUD.

Knowledge competency and skill-based learning are crucial components for workforce development for RCs. Currently, Kentucky peer support certification training is minimal (~30 h). This is likely inadequate to help all clients with recovery needs and could potentially cause unintentional harm. A minority of individuals with OUD have experience with MOUD (38). Thus, RCs without an evidence-informed understanding of MOUD may perpetuate stigma and negative opinions around MOUD to their clients, driving them away from linking to and staying retained in MOUD. Our results demonstrate the importance of assessing competence as some RCs need additional assistance to pass competency checks.

### 5.2 Lessons learned

There are prevalent myths and misconceptions around MOUD and harm reduction that change over time (e.g., Are fentanyl test strips legal to possess?), as well as periodic updates to best practice recommendations and misunderstandings from agencies about the role of the RC (e.g., They are not “sponsors” or “therapists”). Therefore, it is important to have continuing education and regularly scheduled check-ins during supervision. Also, there was a need for training to be continually offered due to the rapid turnover in the RC workforce.

## 6 Limitations

The case studies are a representative snapshot of the RC training experience and may not be generalizable to other settings. Future studies could systematically evaluate knowledge and attitudes toward MOUD and efficacy of the material pre- and post-training and deployment from a larger, more geographically diverse sample. Final data are not yet available on the effectiveness of the linkage and retention program, but we are encouraged by over 70% of the agency sites wanting to continue RC services after the HCS study intervention period. Additionally, while the programs are designed to reduce barriers to beginning or continuing MOUD, many of which are caused by structural barriers such as poverty and racism, the training curriculum does not explicitly address health equity. We plan to add trainings on these topics in the future, especially given the increasing disparities in MOUD access in Kentucky (39).

## 7 Conclusion

The HCS-VOH Linkage and Retention RC training curriculum was created specifically to reduce opioid-involved overdose deaths by developing an RC workforce to assist individuals with OUD to begin and/or continue MOUD. Our novel training equipped RCs with a foundational knowledge of MOUD and skills to address stigma and misconceptions around MOUD. Our training model shows promise as illustrated by the presented case studies and majority of venues across several settings (e.g., syringe service programs, detention centers, and MOUD clinics) desiring to continue employing RCs after the study period ended. Building a well-trained, evidence-informed RC workforce to help support those with OUD entering and continuing in MOUD treatment is critical to ending the opioid epidemic.

## Data availability statement

The original contributions presented in the study are included in the article/Supplementary material, further inquiries can be directed to the corresponding author.

## Ethics statement

The HCS protocol (Pro00038088), which includes the HCS-VOH Linkage and Retention RC Programs, was approved by Advarra Inc., the HEALing Communities Study single Institutional Review Board (IRB). Written informed consent for all individuals receiving HCS services, (e.g., linkage and retention program participants) was waived by the IRB.

## Author contributions

TM: Conceptualization, Visualization, Writing – original draft, Writing – review & editing. AF-B: Conceptualization, Methodology, Writing – original draft, Writing – review & editing. LF: Conceptualization, Methodology, Writing – original draft, Writing – review & editing. SW: Conceptualization, Investigation, Methodology, Writing – review & editing. CC: Project administration, Writing – original draft, Writing – review & editing. DO: Conceptualization, Methodology, Writing – review & editing. AR: Data curation, Visualization, Writing – review & editing. MG: Project administration, Visualization, Writing – review & editing. JL: Project administration, Writing – review & editing. JB: Supervision, Writing – review & editing. PW-C: Conceptualization, Project administration, Writing – review & editing. ML: Conceptualization, Methodology, Writing – original draft, Writing – review & editing.

## Footnotes

^1^ https://healingstudy.uky.edu/sites/default/files/2023-11/HCS-VOH%20RC%20Manual%20FINAL%2011.6.23.pdf
