# Supplementary material for: The development of a recovery coaching training curriculum to facilitate linkage to and increase retention on medications for opioid use disorder
Source: Front Public Health. 2024 Feb 15;12:1334850. doi: 10.3389/fpubh.2024.1334850 (PMC10903364; doi:10.3389/fpubh.2024.1334850)
Supplement: Supplementary file 1 [file Table_1.pdf]

**Supplemental Table 1:** Recovery coach training experience interview guide and sample responses

| Interview Questions & Prompts | Sample Responses |
|---|---|
| Tell me about your training experience for the linkage and retention programs:<br><br>What stood out to you or was memorable?<br><br>What information or topics were new to you? | *"RCA was really interactive… I did peer support prior to Voices of Hope* (VOH)*… RCA was way more beneficial to the work we do here [compared to the state certification]"*<br><br>*"Harm reduction [was new to me]… I didn't know what harm reduction entailed… I always thought harm reduction was… the same as how I was explaining enabling to someone [previously in my AA background]… I just didn't have the knowledge"*<br><br>*"[the Harm Reduction as a Recovery Pathway trainer] asked me, Well do you want [people in active opioid use] to die?' and I said 'No'… so it opened my eyes to look at it in a different light."*<br><br>*"The motivational interviewing* (MI) *training taught me how to do open-ended questions… keep the conversation … [and] broaden the entire conversation"* |
| Tell me about your experience in the MOUD Competency training. | *"[Taking MOUD is] stigmatized… [potential participants] say 'you're not really sober if you're on these medications'… now I can explain the difference between opioid [physical] dependence and opioid use disorder… it's good we go over it in detail like we do."*<br><br>*"Participants use [the flyer] to make a decision… [it is then] easier to make a decision or have an understanding of why MOUD is used"* |
| What did you think about the interactive components of this training? | *"the best [training] experience at any job I've ever had... a lot of workplaces will throw you in but having one-on-one [role plays to practice] was very helpful"*<br><br>*"in other recovery jobs you are taught to be their friend and have to adapt skills on your own… this training taught me a whole other way to talk to people."* |
| Tell me about how your views on MOUD may or may not have changed. | *"whole perspective has changed. I understand addiction better, understand chronic illness better, the disease* |

| | |
|---|---|
| | *process, how the medications work, and see how they help people live successful lives"*<br><br>*"[MOUD] is saving lives, it's keeping people alive … [now I believe] everybody's recovery looks different, whatever works for that person works for them. I have a lot of conversations with other people, like in fellowships, and explain [MOUD] to them."* |
