# Supplementary material for: The development of a recovery coaching training curriculum to facilitate linkage to and increase retention on medications for opioid use disorder
Source: Front Public Health. 2024 Feb 15;12:1334850. doi: 10.3389/fpubh.2024.1334850 (PMC10903364; doi:10.3389/fpubh.2024.1334850)
Supplement: Supplementary file 2 [file Data_Sheet_1.pdf]

HEALing Communities Study KY and Voices of Hope Lexington

Medications for Opioid Use Disorder Linkage and Retention

Recovery Coach Training Manual

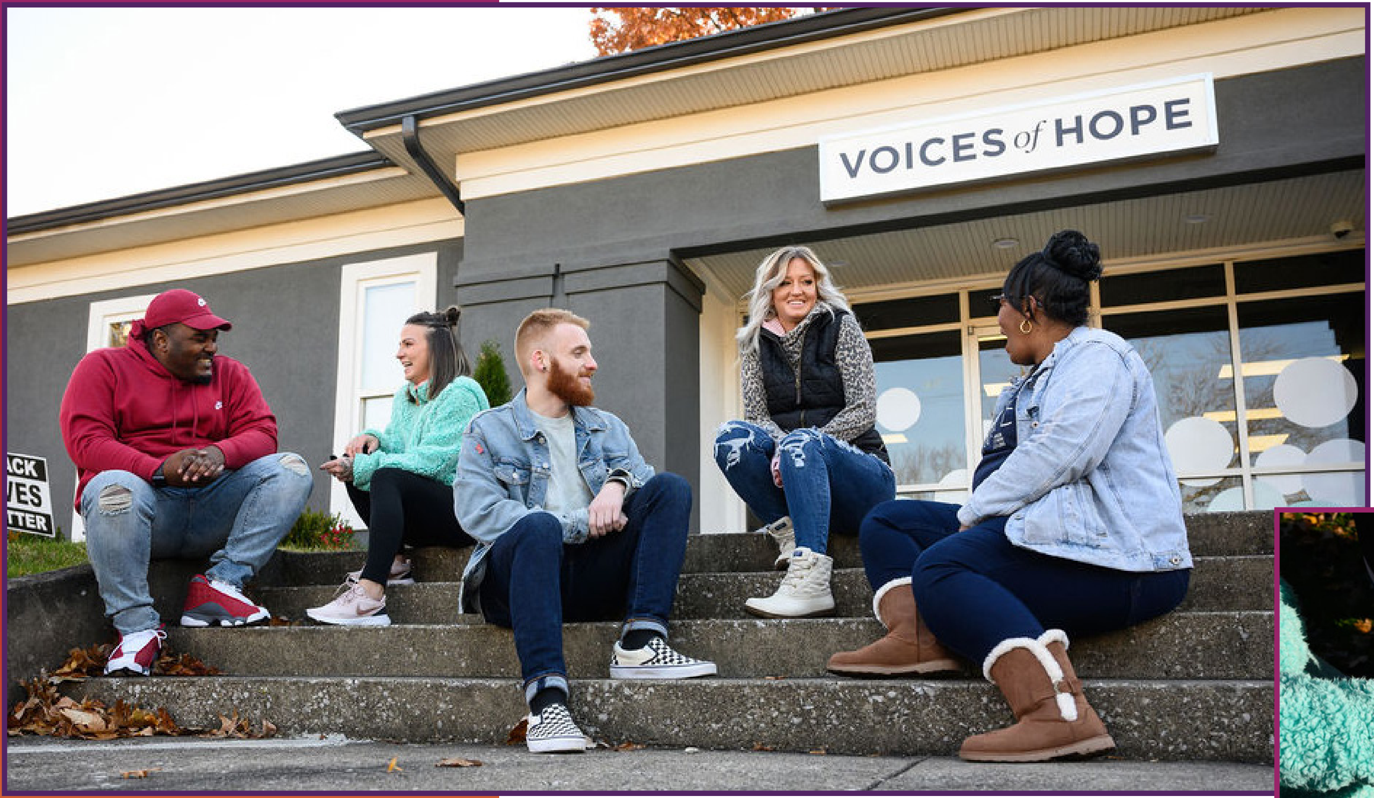

NIH HEAL INITIATIVE

HEALing Communities Study Kentucky

VOICES of HOPE

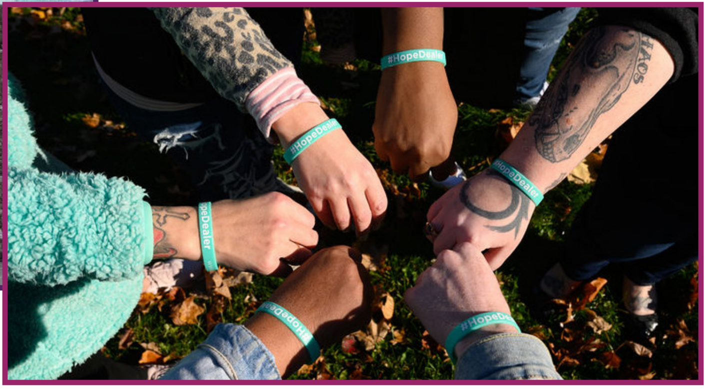

Table of Contents

List of Abbreviations 4

Acknowledgments 5

Introduction 7

- The Need for Linkage and Retention Peer Support Programs and this Training . . 7
- How to Use this Manual 8
- Additional Considerations 9

Domain 1: Orientation and Onboarding 10

- Sample Training Schedule 10
- Linkage and/or Retention Palm Card 10
- Transition Plan Document 10
- VOH-HCS Program Overview 11

Domain 2: Standard VOH RC Training 12

- KY State Adult Peer Support Specialist (APSS) Certification 12
- Connecticut Community for Addiction Recovery (CCAR) Recovery Coach Academy (RCA) 12
- Question, Persuade, Refer (QPR) Suicide Prevention Training 13
- Basic Life Support (BLS) Training 13
- Human Subjects Protection (HSP) Training 13
- Self-Management and Recovery Training (SMART) Recovery & SMART Family & Friends (F&F) 14
- Telephone Recovery Support (TRS) Didactic Training and Shadow Shift 14
- Harm Reduction as a Pathway of Recovery 15

Domain 3: SUD and MOUD Educational Trainings 16

- ***Part 1: Providers Clinical Support System (PCSS) SUD 101 Modules 16***
- Changing Language to Change Care: Stigma and Substance Use Disorder . . .17
- Overview of Substance Use Disorders 17

Screening, Assessment, and Treatment Initiation for SUD 17
Medications for Opioid Use Disorder 17
Principles of Motivational Interviewing: Useful for Primary Care Providers 18
Introduction to the Criminal Justice System and MOUD 18
Preventing Opioid-Involved Overdose with Education and Naloxone 18

Part 2: Novel HCS-Developed Trainings 18
MOUD: What Clients Ask Peers 18
Orientation to MOUD Educational Flyer and MOUD Flyer Training 19
Orientation to Community Resource Guides (CRGs). 21
Introduction to Criminal Legal System (CLS) Training 21
Overview of Court Processes and Custodial Changes 22
Orientation to Opioid Treatment Programs (OTPs). 22
A Discussion on Boundaries 22
OUD Regulatory Training for Peers 23

Part 3: KY Opioid Overdose Prevention Education Network (KY-OPEN) Core Sessions. 23
SUDs and Other Behavioral Health Screening and Assessment. 23
MI Part 1: Skills and Strategies (Didactic) 24
MI Part 2: Q&A/Workshop 24
Harm Reduction in Healthcare and Beyond 24

Domain 4: SOP Reviews 25
Linkage and Retention SOP 25
Data SOP 25

Domain 5: Overdose Education and Naloxone Distribution (OEND) . . . 26
Overdose Education Video and Training 26
Overdose Education Tri-Fold Brochure 26

Domain 6: Ongoing Education 27

References. 28

List of Abbreviations

This glossary is a quick reference for abbreviations and acronyms used in this manual.

CEU .... Continuing Education Units
CJ .... Criminal Justice
CLS .... Criminal Legal System
EBP .... Evidence-Based Practice
HCS .... HEALing Communities Study
HCS-KY .... Kentucky site of the HEALing Communities Study
IRB .... Institutional Review Board
KY .... Kentucky
LRC .... Lead Recovery Coach
MI .... Motivational Interviewing
MOUD ..... Medication for Opioid Use Disorder
OBOT ...... Office-Based Opioid Use Disorder Treatment
OEND ...... Overdose Education and Naloxone Distribution
OTP .... Opioid Treatment Program
OUD .... Opioid Use Disorder
PCSS ...... Providers Clinical Support System
PSS .... Peer Support Specialist
RC .... Recovery Coach
ROI .... Release of Information
SMART ..... Self-Management and Recovery Training
SOP .... Standard Operating Procedure
SUD .... Substance Use Disorder
UK .... University of Kentucky
VOH .... Voices of Hope

Acknowledgments

Funding

This training manual was developed as part of the Kentucky site's implementation of the National Institutes of Health (NIH) HEAL (Helping to End Addiction Long-Term[SM]) Initiative, which was supported by the NIH and the Substance Abuse and Mental Health Services Administration through the NIH HEAL Initiative[SM] award number, UM1DA049406, (ClinicalTrials.gov Identifier: NCT04111939). This study protocol (Pro00038088) was approved by Advarra Inc., the HEALing Communities Study single Institutional Review Board. The content is solely the responsibility of the authors and does not necessarily represent the official views of the National Institutes of Health, the Substance Abuse and Mental Health Services Administration, or the NIH HEAL Initiative[SM].

Principal Developers of the Linkage and Retention Programs

Amanda Fallin-Bennett, Ph.D., RN
KY HCS Community Engagement Team Faculty
Associate Professor, University of Kentucky (UK) College of Nursing
Co-Founder, VOH
amanda.fallin@uky.edu

Laura Fanucchi, MD, MPH
KY HCS Treatment Team Lead
Associate Professor, Division of Infectious Diseases
Director, Addiction Consult and Education Service
UK College of Medicine (COM)
UK Center on Drug and Alcohol Research (CDAR)
laura.fanucchi@uky.edu

Michelle Lofwall, MD, DFAPA, DFASAM
KY HCS Treatment Team Lead
Professor, Departments of Behavioral Science and Psychiatry
Bell Alcohol and Addiction Chair
Medical Director, First Bridge and Straus Clinics
UK COM
UK CDAR
michelle.lofwall@uky.edu

Devin Oller, MD, FACP, FASAM
KY HCS Treatment Team Faculty
Assistant Professor, Department of Internal Medicine
Associate Program Director, Internal Medicine Residency Program
UK COM
devin.oller@uky.edu

Training Development and Implementation

Special thanks to our Principal Investigator, Treatment Team, Implementation Science Team, Criminal Legal System Team, Project Directors, and HCS and VOH staff who also helped develop and support the training program, barrier relief programs, ongoing continuing education, standard operating procedures, and tools for data collection for program monitoring and evaluation contained within this manual.

Candace Brancato, MS
Data Scientist
UK College of Public Health
Department of Biostatistics
candace.brancato@uky.edu

Christopher Cook, MS
KY HCS Treatment Program Manager
UK Substance Use Priority Research Area (SUPRA)
chris.cook@uky.edu

Molly Gallivan, MPH, CPH
KY HCS Treatment Program Coordinator
UK SUPRA
molly.gallivan@uky.edu

Brooke Hiltz, JD
KY HCS Community Program Manager
UK SUPRA
brooke.hiltz@uky.edu

Hannah Knudsen, Ph.D.
KY HCS Implementation Science Team Lead
Professor, Department of Behavioral Science
UK COM
UK CDAR
hannah.knudsen@uky.edu

Liz Larimore, MS
KY HCS Associate Director
UK SUPRA
elizabeth.larimore@uky.edu

John Lauckner, MA
KY HCS Treatment Education Coordinator
UK SUPRA
john.lauckner@uky.edu

Margaret McGladrey, Ph.D.
KY HCS Criminal Legal System Team Lead
Research Assistant Professor, Department of Health Management and Policy Center for Innovation in Population Health
UK College of Public Health
margaret.mcgladrey@uky.edu

Jennifer Miles, CPA
KY HCS Executive Director
UK SUPRA
jennifer.miles@uky.edu

Trevor Moffitt, Ph.D.
KY HCS Community Engagement & Recovery Services Administrator
UK SUPRA
trevor.moffitt@uky.edu

Carrie Oser, Ph.D.
KY HCS Criminal Legal System Team Lead
Di Silvestro Endowed Professor, Department of Sociology
University Research Professor
Associate Director, Center for Health Equity Transformation
UK College of Arts and Sciences
UK CDAR
carrie.oser@uky.edu

Anna Ross, BS, APSS
KY HCS Data Manager, VOH
anna@voicesofhopelex.org

Sharon L. Walsh, Ph.D.
Principal Investigator HEALing Communities Study, Kentucky
Professor of Behavioral Science, Psychiatry, Pharmacology, and Pharmaceutical Sciences
Director, UK CDAR
sharon.walsh@uky.edu

We wish to acknowledge our peer recovery support partner agency, **Voices of Hope (VOH)**, and their staff as well as community agencies who welcomed implementation of MOUD linkage and retention utilizing recovery coaches (RC)s at their site.

We also want to acknowledge the participation of the HEALing Communities Study communities, community coalitions, and Community Advisory Boards and state government officials who partnered with us on this study.

Introduction

The Need for Linkage and Retention Peer Support Programs and this Training

The opioid epidemic continues with over 100,000 opioid-related overdose deaths reported in the United States in 2021, a 59% increase since 2019 (Ahmad et al., 2023). Medications for opioid use disorder (MOUD), specifically methadone and buprenorphine, are standard care opioid use disorder (OUD) treatments. They reduce opioid-involved overdose and all-cause mortality (LaRochelle et al, 2018; Wakeman et al., 2020) and are associated with decreases in illicit opioid use, property crime, and infectious disease transmission (National Academies of Sciences, Engineering, and Medicine, 2019). Unfortunately, as many as 86% of individuals who may benefit from MOUD do not receive it (Krawczyk et al., 2022).

The HEALing (Helping to End Addiction Long-term℠) Communities Study (HCS) is a community-engaged research intervention with a primary aim to reduce opioid overdose deaths by 40% (Walsh et al., 2020). The HCS intervention, Communities that Heal (CTH), seeks to implement evidence-based practices (EBP) focused on overdose education and naloxone distribution (OEND); increasing access, linkage, and retention in MOUD; and increasing prescription opioid safety. One strategy used in KY to assist with linkage and retention in MOUD was to employ peer support specialists (PSSs; persons with lived experience of substance use disorder) and train them to become recovery coaches (RCs) with a critical skillset tailored to working specifically with persons who use opioids and employ several tools (e.g., recovery coaching, motivational interviewing (MI), barrier relief funding, etc.) to help persons link to and stay retained in MOUD. RCs are a type of peer worker who are in remission and recovery and perform non-clinical recovery support services like facilitating goal-setting with participants, advocating for individuals with SUD, making referrals to resources, and inspiring hope that remission and recovery are possible (Jack et al., 2018).

Even though I used MOUD as part of my own recovery before coming to work with HCS, the training helped me better understand how MOUD works and helps me explain this to others. I also realized just how stigmatized MOUD is, and the training helped me be prepared to address the stigma to participants.

HCS-VOH KY Trained RC

Evidence suggests that those who use drugs prefer to work with peers who share their experience and often learn about treatment options from them, such as MOUD (Bardwell et al, 2018). Recovery coaching for participants with OUD is novel and shows promise in facilitating MOUD linkage and retention and decreasing risk for overdose (Gormley et al., 2022, Winhusen et al., 2020). However, there are scant standardized training and implementation practice manuals focused on ensuring adequate health literacy among RCs about OUD and MOUD treatments or skills/tools needed to assist in resolving barriers to linking and retaining on MOUD. Current statewide peer support certifications are often received after completing a one-time peer training program with occasional ongoing education (Dahlem et al., 2021; Magidson et al., 2021). Thus, the HCS-KY research team partnered with VOH to develop two novel RC training programs — the MOUD linkage program and the MOUD retention program.

The purpose of this manual is to share the content and training resources developed by the HCS-KY team to implement the linkage and retention RC programs. The linkage program aims to link individuals with OUD in various settings (e.g., syringe support programs) to MOUD treatment. The retention program aims to engage with individuals already in MOUD treatment (e.g., licensed opioid treatment programs) who may be at risk for dropping out.

RC Training Plan

How to Use this Manual

The RC training manual is divided into six sections: (1) **Orientation and Onboarding**, (2) **Standard VOH RC Training**, (3) **SUD and MOUD Educational Trainings**, (4) **SOP Reviews**, (5) **OEND**, and (6) **Ongoing Education**. Each training component can be accessed by clicking on the relevant title in the table of contents. A list of training requirements is also summarized in the **RC Training Plan**, which serves as a checklist for the RC to mark off trainings as they are completed. A manuscript describing the training programs is currently under review.

Each training component in this manual is listed as a title. Information below each title includes the length and format of the training and a brief summary of the training. Materials related to training components are included as links in the sidebar to the left of the training information. Most of the training resources are available via a hyperlink. Note some links may be a file download.

Additional Considerations

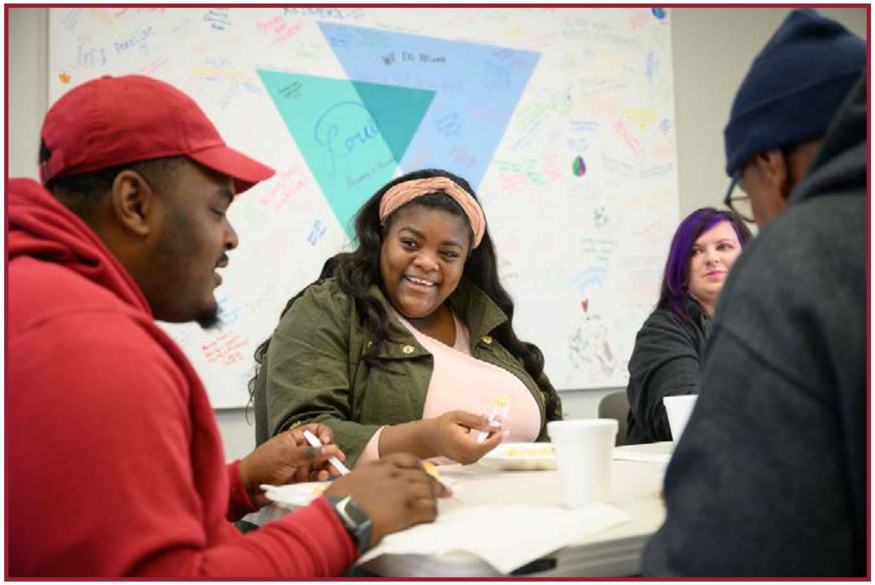

Many training components of the RC program are unique to HCS and our recovery support services community partner, VOH, or the way we have chosen to implement various components of our programs. For example, there are standard operating procedures (SOPs) on the way that we handle administrative aspects of the RC role [e.g., setting up a Microsoft Teams Account, getting staff badges] and lines of communication between the RC and other teams involved in the study. We have not included some of these components because they are unlikely to be helpful to other organizations. In some cases, we described trainings and explained that SOPs or other materials further supporting that aspect of training/program implementation are available upon request. Please note that the training manual information contained herein may not be directly applicable to other organizations due to various issues such as local and state differences in regulation and policy (e.g., state variation in requirements for OEND). Regardless of these differences, we hope this manual provides a sample and scope of the potential resources that may be needed to successfully implement peer support training programs aimed at helping people with OUD link to and stay retained in MOUD treatment.

I want to share a success story of one of my participants. I started working with this participant about two months ago. When I first met him, he was [experiencing homelessness], without family, without friends, and he felt like he did not have any direction in life or hope. Today, he has not used in one month. We were able to put him into emergency housing for a week. He has now transitioned to a sober living facility. We got an ID made for him, got treatment, set up interviews for employment, and he will start his new job this coming Monday.

HCS-VOH KY Trained RC

Domain 1

Orientation and Onboarding

The following information assists individuals in orienting to their role of a linkage or retention RC. This section provides an overview to the linkage and retention RC programs and important administrative onboarding requirements.

VOH Training Schedule

Sample Training Schedule

Document review; approximately 10 minutes

When organizations choose to implement this training manual, an onboarding schedule with trainings and timeframes for completing them will aid in orienting and providing guidance to the RC role. A sample onboarding training schedule is provided.

VOH Linkage Palm Card Template

VOH Retention Palm Card Template

VOH Linkage/Retention Combination Palm Card Template

Linkage and/or Retention Palm Card

Live, online, or in-person; approximately 10 minutes

Palm cards are similar to business cards. The 3x5 cards provide an overview of the linkage or retention programs as well as a picture and phone number of the RC so that participants/potential participants can review the information and have a tangible reference to get in touch with RCs as needed. Sample templates for the linkage and retention program palm cards are provided.

Transition Plan Template

Transition Plan Document

Live, online, or in-person; approximately 10 minutes

The Transition Plan is a form designed to help RCs and their supervisors understand the key persons/points of contact at the agency where they will be deployed temporarily (e.g., to help cover for a RC who is out sick, on vacation, etc.) or permanently (e.g., previous RC is leaving their position). The document summarizes relevant information about the specific agency and is meant to facilitate onboarding. We recommend that RCs deploying to community agencies create or update transition plans to facilitate continuity and consistency among operations at community partner sites. A template for completing transitions plans is provided.

- **VOH-HCS Linkage and Retention Recovery Coach Program Overview**
- **Peer Support Services for People with OUD: Linkage and Retention Programs for MOUD**

VOH-HCS Program Overview

Live, online, or in-person; approximately 2 hours

This training provides an in-depth overview of the HCS linkage and retention programs for RCs across the OUD care continuum including pre-linkage, linkage, treatment initiation, and retention services. The linkage and retention programs are strengths-based, person-centered, and designed to build recovery capital to support life-long remission and recovery from OUD. Remission means no longer having any symptoms of OUD. Recovery means a process of change through which individuals improve their health and wellness, live self-directed lives, and strive to reach their full potential.

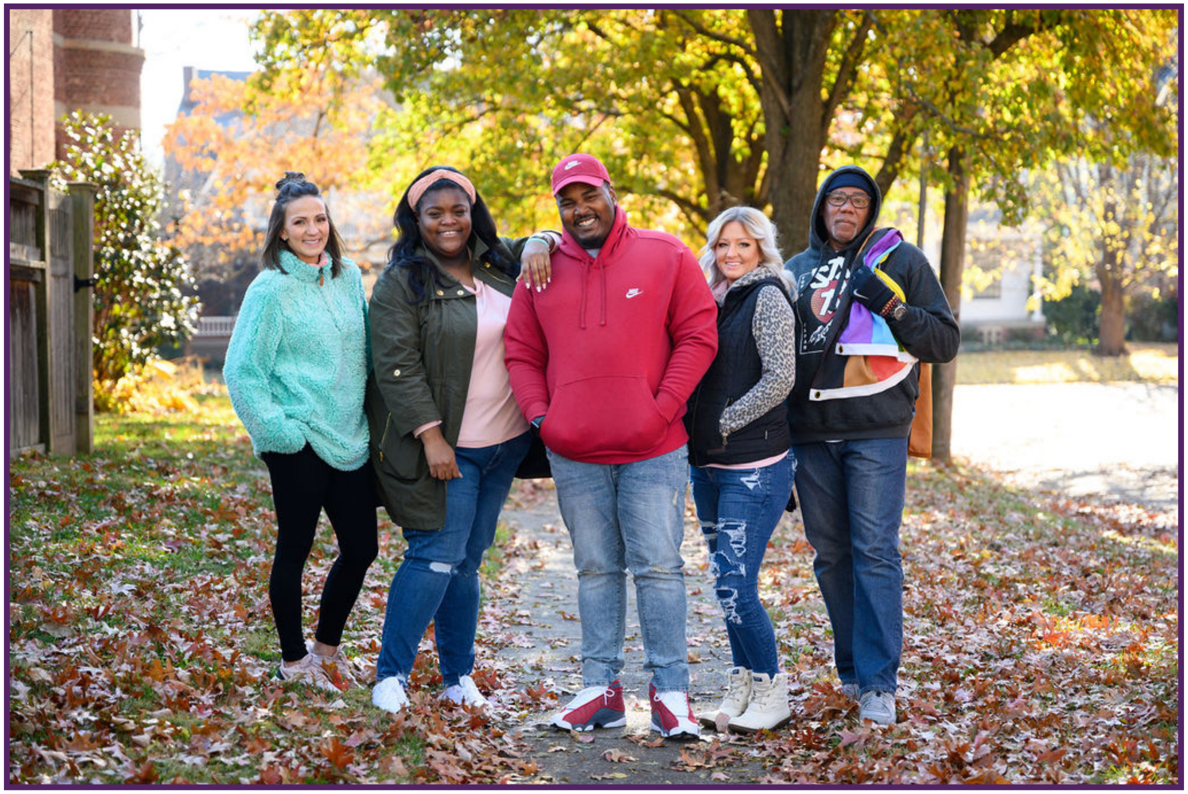

Domain 2

KY DBHDID Adult Peer Support Specialist Approved Curricula Contact Information

908 KAR 2:220 Kentucky Administrative Regulation Regarding Peer Support Specialists

CCAR Trainings Curriculum

(See Recovery Coach Academy information listed first on page)

Standard VOH RC Training

RCs who are part of HCS complete all training that is standard to our recovery support services community partner organization, VOH. The standard VOH RC training consists of training in the basics of peer support services and recovery coaching (including a RC academy training that expands upon state required PSS certification), locally available community resources, key principles of harm reduction, and participant activation and engagement training. We recommend that all organizations implementing RC linkage and retention programs ensure that all RCs are trained in similar content.

Please note our team also provides a training regarding securing appropriate releases of information (ROI) from participants and community partners. However, our ROI forms are not included below because they are partner specific. We recommend agencies ensure that HIPAA/42 CFR Part 2 ROI consent forms and training are provided to RCs.

KY State Adult Peer Support Specialist (APSS) Certification

Live, online, or in-person; minimum 30 hours

The KY Department for Behavioral Health, Development, and Intellectual Disabilities (DBHDID) established certification criteria for peers with approved curricula and ongoing continuing education units (CEUs) requirements. Each state may have their own certification criteria. The materials provided describe the KY requirements. Peers who are not already certified upon hire go through this training as part of their onboarding process.

Connecticut Community for Addiction Recovery (CCAR) Recovery Coach Academy (RCA)

Live, in-person; approximately 30 hours

HCS peers become certified RCs to supplement their state-based APSS training and knowledge of the peer support role, principles of recovery, wellness planning, boundaries, and practice of MI techniques and effective use of self-disclosure.

QPR Institute

Question, Persuade, Refer (QPR) Suicide Prevention Training

Live, online, or in-person; 1 hour

Many RCs may be new to working with individuals who may experience suicidal ideation. This training dispels myths about suicide and helps RCs understand and recognize warning signs, including how to respond to someone who is potentially suicidal.

Note: Many National Alliance on Mental Illness (NAMI) chapters offer QPR training at no cost. Contact your local NAMI chapter for details.

American Heart Association Basic Life Support Course

American Red Cross BLS Certification Course Finder

Basic Life Support (BLS) Training

Live, in-person; approximately 4 hours
Self-paced, online; approximately 2 hours

RCs complete BLS training to learn how to respond to medical emergencies they may encounter while working in the field.

Association of Clinical Research Professionals (ACRP) Human Subject Protection Training

Human Subjects Protection (HSP) Training

Self-paced, online; approximately 4 hours

Since RCs are working with HCS, a human subject research study whereby they are collecting data from the linkage and retention program participants, they must complete HSP training. This training provides a consolidated overview on the history and importance of ethical conduct in human research. This HSP training is recognized by the University of Kentucky Institutional Review Board (IRB) and allows RCs to be added to the IRB as key personnel following training completion. The training covers important concepts of beneficence, nonmaleficence, autonomy, and justice, which are also concepts discussed during the boundaries training. We encourage other programs to prospectively collect clinical data and thus encourage HSP training.

Note: This training is offered by ACRP for community members engaged in the research process; RCs register for Ethics and Human Subjects Protection: A Comprehensive Introduction. This course is provided at no cost, but there are no CEUs available for the free course.

Combined SMART and SMART F&F Training Information

Self-Management and Recovery Training (SMART) Recovery & SMART Family & Friends (F&F)

Self-paced, online; approximately 40 hours

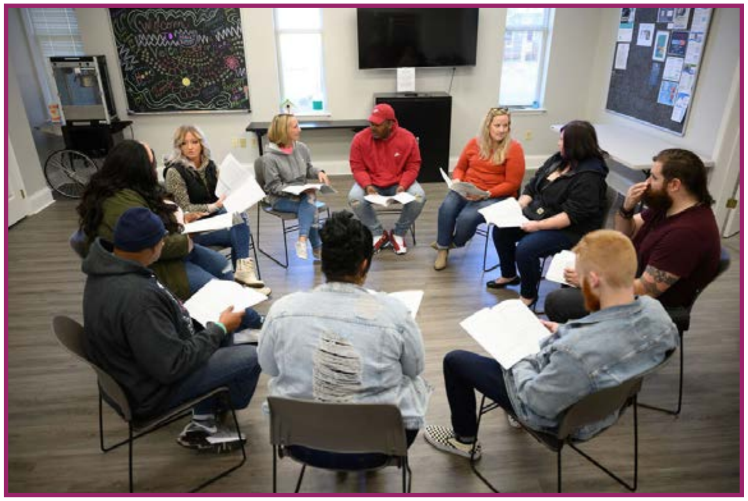

Another job duty of a HCS RC is to lead groups utilizing the SMART Recovery program. SMART Recovery is an evidence-based recovery support program utilizing “4-points” that empower individuals to design and implement their own recovery plan. At the end of the training, RCs take a written exam and shadow a SMART meeting led by a fellow RC prior to leading their own meetings.

Sample TRS Calls

TRS Crisis Response Protocol for Suicide Risk

Telephone Recovery Support (TRS) Didactic Training and Shadow Shift

Live, in-person; approximately 2 hours

VOH manages a TRS line as an integral part of the HCS retention program. RCs conduct TRS calls, connect participants to resources, and provide support for the participant and their recovery journey. This service is an additional support to the individual recovery coaching that participants receive from their assigned RC. During this training, RCs listen to other experienced RCs take calls and practice sample scripts.

VOH Harm Reduction Training Presentation

Infographics

- Opioid Overdose
- Wound Care
- Cleaning Equipment
- Safer Injection
- Safer Smoking Tips
- Never Use Alone

Harm Reduction as a Pathway of Recovery

Live, in-person; 4 hours

This training provides an overview of Harm Reduction as a pathway of recovery with discussion of practical harm reduction strategies and importance of changing language to address stigma surrounding people who use drugs. All materials are evidence-based materials adapted from the National Harm Reduction Coalition, a leader in harm reduction efforts. Infographics/flyers are also used throughout this training, and RCs are encouraged to integrate them into their work with participants when educating on harm reduction techniques.

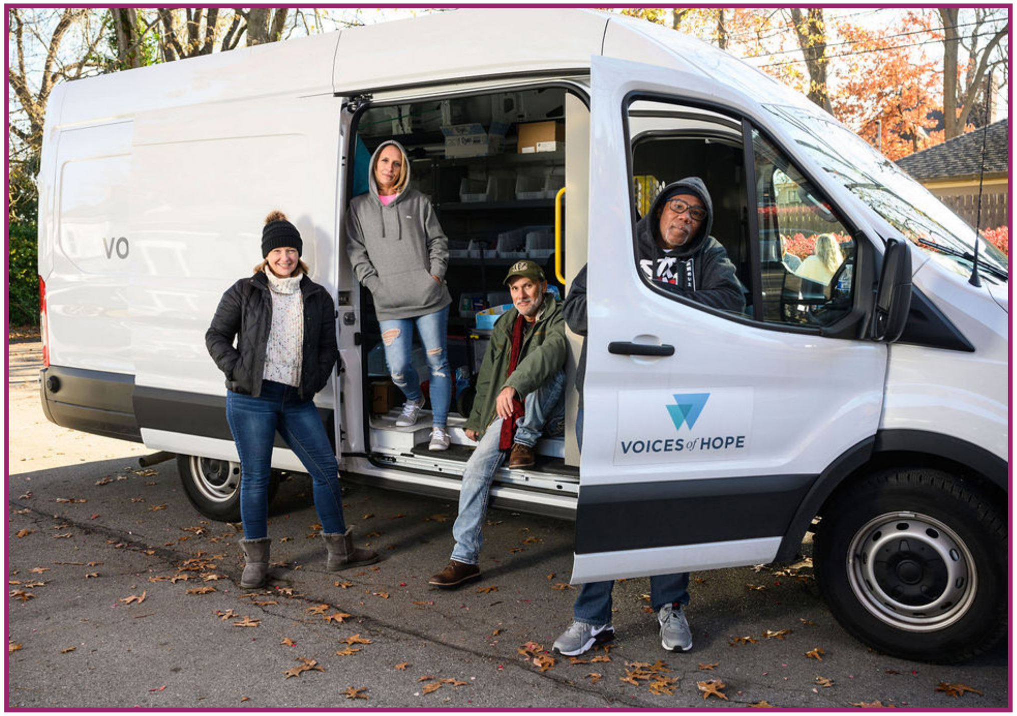

Domain 3

SUD and MOUD Educational Trainings

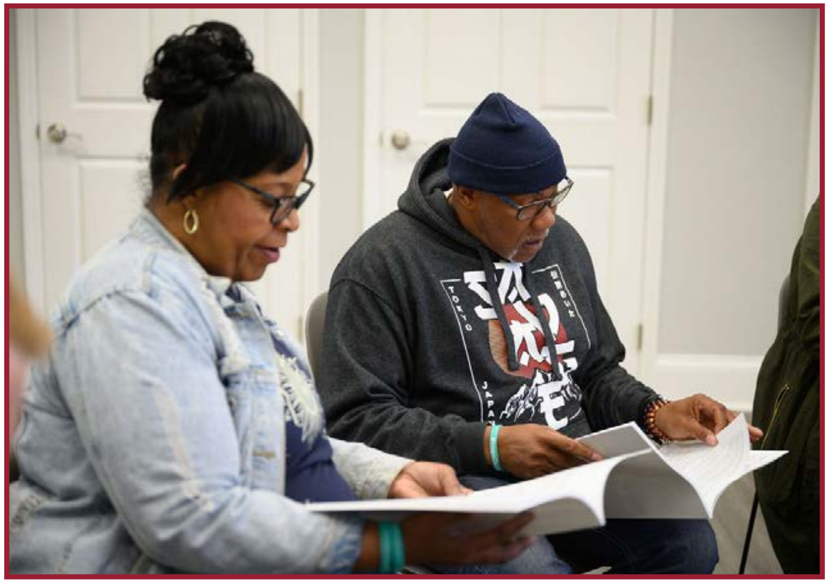

To successfully implement the OUD linkage and retention programs, all RCs must have a foundational knowledge of SUD and more in-depth knowledge about OUD and its FDA- approved medication treatments. This education is critical to understanding some of the concerns that participants may have about entering or remaining in MOUD treatment. This training prepares RCs to answer questions about medication, identify symptoms of craving and withdrawal, and help dispel myths and misunderstandings around OUD and its treatments.

The following set of trainings are intended to provide an evidence-based foundation to understand OUD (and other SUDs); critical medication treatments for OUD, specifically methadone and buprenorphine; and effective communication and MI skills relevant to working with persons considering treatment with MOUD.

SUD 101 Core Curriculum (2023)

Part 1: Providers Clinical Support System (PCSS) SUD 101 Modules

The PCSS SUD 101 modules provide an overview of EBPs in the prevention, identification, and treatment of SUDs and co-occurring mental disorders. RCs who complete these modules should see an increase in their knowledge as they assist participants with their own SUD. All PCSS SUD 101 modules are available online at **SUD 101 Core Curriculum (2023)**. Please note RCs will be required to create an account to access materials. Upon completing a module, the RC earns a certificate of completion. RCs should save each of their certificates for training and continuing education units (CEU) documentation. When completing the CEU forms, the RC selects the credit that applies best to them and their role.

Changing Language to Change Care: Stigma and Substance Use Disorder Presentation

Changing Language to Change Care: Stigma and Substance Use Disorder

Self-paced, online; approximately 1 hour

This training provides an understanding of the importance of language when discussing substance use and reviews ways to improve language and reduce stigmatizing language to improve care.

My background from 12-step programs led me to use terms like 'clean' and I thought people had to be 'sober' or 'abstinent' to really be in recovery. This and other trainings have broadened my horizon and vocabulary to use more inclusive language.

HCS-VOH KY Trained RC

Overview of Substance Use Disorders Presentation

Overview of Substance Use Disorders

Self-paced, online; approximately 1 hour

This training provides a foundation in knowledge of SUD including the spectrum of use, neurobiological responses to substances, theories that explain the disorders, public health impact and epidemiology, comorbidity, and integrated care for SUDs.

Screening, Assessment, and Treatment Initiation for SUD Presentation

Screening, Assessment, and Treatment Initiation for SUD

Self-paced, online; approximately 1 hour

This training introduces screening tools for drug and alcohol use, reviews detailed medical and psychiatric assessment considerations, and discusses considerations for treatment referrals.

Medications for Opioid Use Disorder Presentation

Medications for Opioid Use Disorder

Self-paced, online; approximately 1 hour

This training provides a foundational understanding of the evidence for each FDA-approved MOUD and compares each medication. It also includes discussion of a case vignette in which MOUD options are considered.

Principles of Motivational Interviewing: Useful for Primary Care Providers Presentation

Introduction to the Criminal Justice System and MOUD Presentation

Preventing Opioid-Involved Overdose with Education and Naloxone Presentation

MOUD: What Clients Ask Peers Presentation

Principles of Motivational Interviewing: Useful for Primary Care Providers

Self-paced, online; approximately 1 hour

This training introduces key MI principles, four key processes, the spirit of MI, patient-centered communication skills, and the importance of evoking change talk.

Introduction to the Criminal Justice System and MOUD

Self-paced, online; approximately 1 hour

This training provides a foundational understanding of the criminal legal system and how to help individuals involved throughout this system from arrest to incarceration to re-entry.

Preventing Opioid-Involved Overdose with Education and Naloxone

Self-paced, online; approximately 1 hour

This training provides an overview of overdose prevention education and prescribing naloxone to people at risk for opioid overdose.

Part 2: Novel HCS-Developed Trainings

As part of the onboarding process, RCs complete a number of trainings developed by the HCS team to aid in their understanding of OUD, MOUD, and educating potential participants as part of their RC role.

MOUD: What Clients Ask Peers

Self-paced, online; approximately 30 minutes

This training provides an overview of MOUD. It is specifically designed for RCs including common questions from participants about MOUD and suggested responses.

MOUD Flyer

MOUD Flyer Training Checklist

MOUD Checklist Grading Rubric

Orientation to MOUD Educational Flyer and MOUD Flyer Training

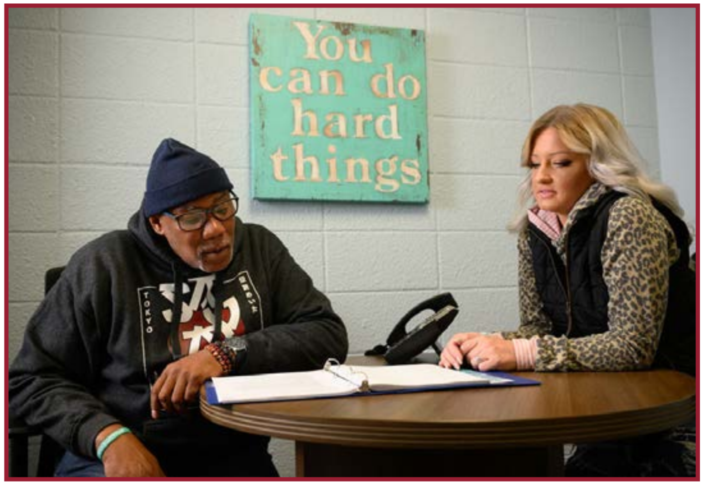

Live, in-person or self-guided, online; approximately 22 hours

HCS created a **MOUD Educational Flyer** that is intended to help improve health literacy around MOUD for all communities, including RCs. The goal of the MOUD flyer training process is to improve the RC workforce health literacy around MOUD so that RCs can effectively share this knowledge with participants, help participants learn about OUD and its medication treatments, and prevent the spread of misinformation. RCs use this new knowledge along with their own lived experience and other critical skills (e.g., MI, maintenance of appropriate boundaries, recovery capital assessment, etc.) to provide hope and engage with participants.

MOUD Flyer Orientation Video

Step 1: Prerequisite Training

Self-paced, online; approximately 45 minutes

Before RCs engage in live MOUD flyer training, they should acquaint themselves with the MOUD flyer as well as the training checklist and the grading rubric used to verify competency at the end of the training. A video provides RCs with an orientation to the MOUD flyer and how it should be used with participants.

Note: The HCS-VOH program also incorporates internal use only videos where trainers and RCs roleplay mock scenarios that RCs in training can watch before participating in live training. Other organizations may find it helpful to include similar videos in their training programs.

Step 2: Building Foundational Knowledge with Experienced Peer

Live, in-person or online; approximately 2 hours

Following the video introduction, the RC will have a basic understanding of the MOUD Flyer. This training builds on that, providing foundational knowledge and consists of two separate one-hour training sessions. The first session provides an overview of the flyer and each section of content. The second session will introduce the RC to the role play scenarios that will be a part of the final competency. This training is conducted by an experienced peer, a Lead RC (LRC).

“I only knew one person who had been on methadone, and before this training I always judged it based on that person’s experience and wasn’t sold on the value of MOUD. I thought MOUD were okay but realized I didn’t actually know much about them. Now I see that MOUD helps keep people alive and allows them to live self-directed lives.”

HCS-VOH KY Trained RC

- **A Taste of Motivational Interviewing Slides**
- **HCS Recovery Coach MOUD Flyer Roleplay Checklist**
- **MOUD Generating Helpful Responses Worksheet**

Step 3: Basics of Motivational Interviewing, Presentation of MOUD Flyer, and Common MOUD Questions

Live, in-person; approximately 8 hours

This training provides a foundational overview of MI to inform how to use the MOUD flyer and its components. The training consists of an overview of MI by a Motivational Interviewing Network of Trainers (MINT) trainer, an overview of flyer content by a peer recovery program director, and an application of the MI skills to the MOUD flyer content and case study examples.

Step 4: Practicing with the MOUD Flyer: Mock Participant-RC Discussions and Teach-Back Training

Live, in-person; approximately 8 hours

This intensive training practice prepares RCs to use the MOUD flyer as an educational tool in linkage and retention participant scenarios. The RC practices with an experienced RC and/or supervisor and applies their learned MI skills during a full mock recovery coaching session that involves demonstration of MOUD flyer knowledge and linkage and retention programs (e.g., frequency of contacts, risk assessment). The day-long training is facilitated by a MINT certified trainer.

Step 5: Final Prep Sessions with Experienced Peer

Live, in-person or online; approximately 2–4 hours

After completing the previous four steps, RCs now meet with a VOH LRC to prepare for the final MOUD Flyer competency review. Practice sessions are held in different formats, and the content focus and number of times the RC practices is tailored to meet individual RC needs.

MOUD Flyer Training Checklist

MOUD Checklist Grading Rubric

Orientation to Community Resource Guides Video

Sample Community Resource Guide (Boyd County KY)

Introduction to CLS Slides

Video: HCS Intro to CLS Training

Criminal Legal System Points of Entry: Sequence of Events in CLS System

Step 6: MOUD Flyer Competency Checklist Completion

Live, online; approximately 45 minutes

Once both the RC and LRC are comfortable with the RC's MOUD flyer knowledge, the final MOUD Flyer competency review is led by the VOH lead reviewer in partnership with a study physician, who together determine if the RC passes this final assessment or needs further training before deployment. Passing/failing is determined using the **MOUD Flyer Training Checklist** and **MOUD Checklist Grading Rubric**.

Orientation to Community Resource Guides (CRGs)

Self-paced, online; approximately 10 minutes

This training describes the CRGs that were created through HCS-KY to facilitate MOUD linkage and retention. Please note that while these resource guides were created for specific communities in KY, this training may be applicable to help other communities create and utilize similar resources to address their participants' needs.

Introduction to Criminal Legal System (CLS) Training

Self-paced, online; approximately 1 hour

These trainings provide an overview of the relationship between OUD and the CLS, acquaint RCs to the CLS venues in which they may work, and provide context for education and working with participants who may be involved in the CLS. Please note that these materials are specific to KY local and state organizations but may still provide useful information for other organizations.

Note: As of August 2022, the KY HCS "criminal justice" team changed its name to the "criminal legal system" team. The materials above were created prior to this change and thus utilize "criminal justice/CJ" terminology.

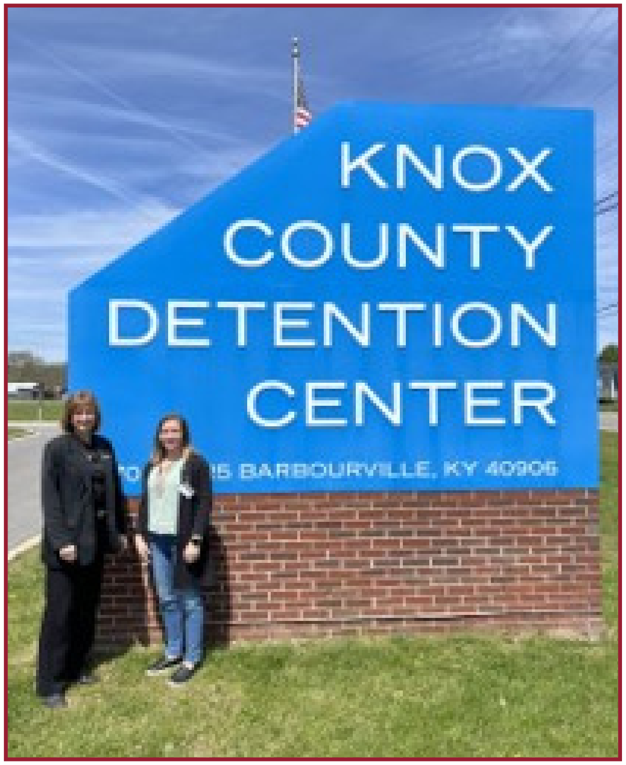

The Adjudication Process in KY's Criminal Justice System

Overview of Court Processes and Custodial Changes

Self-paced, online; approximately 30 minutes

The goal of this training is to explain when and how participants may experience a pre- and post-sentencing change in custody during the adjudication process. This training will help identify key court hearings where changes might be made to aid in anticipating participant release dates in preparation for linkage. The training also helps RCs understand the importance of coordinating with participants to communicate their treatment needs to their public defender/alternate sentencing workers pre-release or parole officer post-release. Please note that this is specific to KY, so other states may need to adapt.

Sample OTP Guides: Welcome to BHG

Orientation to Opioid Treatment Programs (OTPs)

Live, online, and in-person; approximately 1 hour

To understand the resources and service of OTPs, RCs working with these settings have an opportunity for a tour of one of the programs. All RCs also have access to OTP guides for each community, which are one-page documents that explain how RCs can help participants gain rapid access to methadone treatment and share key facts about OTPs that RCs may want to explain to prospective participants.

HCS Recovery Coaches: A Discussion on Boundaries Slides

A Discussion on Boundaries

Self-paced, online; approximately 2 hours

As RCs work with individuals with OUD in the field, issues around creating and maintaining healthy professional boundaries will arise. This training was created in response to direct feedback from our partnering agency with situations that had been brought to their attention from RCs.

OUD Regulatory Training for Peers Video

OUD Regulatory Training for Peers

Self-paced, online; approximately 20 minutes

This training provides a basic overview of MOUD treatment regulations, policies, and laws. At the end of the training, RCs should be able to describe regulatory differences between OTP and non-OTP OUD office-based treatment settings (OBOTs); describe basic requirements of methadone treatment (e.g., visit frequency); and confidentiality protections when working with participants. The training covers informed consent, HIPAA, 42 CFR Part 2, and regulations for OTPs and OBOTs that are important for RCs to understand when linking participants to treatment and helping them stay in MOUD treatment.

Note: Some regulatory aspects have changed and are not yet reflected in this training (e.g., removal of Drug Enforcement Agency "X-waiver"), and some are specific to KY.

Part 3: KY Opioid Overdose Prevention Education Network (KY-OPEN) Core Sessions

KY-OPEN is a weekly teleconference led by OUD clinical experts. Sessions review key OUD topics and provide participants an opportunity to ask questions and discuss issues they are seeing in their communities regarding OUD treatment. Prior to deployment, the KY HCS team requires all RCs to watch the following previously recorded KY-OPEN sessions, because the content is helpful for the RC role and supports their professional development.

Substance Use Disorders and Other Behavioral Health Screening and Assessment

SUDs and Other Behavioral Health Screening and Assessment

Self-paced, online; approximately 1 hour

This training reviews commonly used SUD and Behavioral Health screening and assessment tools for various populations and learn how to integrate screening and assessment tools into workflows.

Motivational Interviewing Skills and Strategies

MI Part 1: Skills and Strategies (Didactic)

Self-paced, online; approximately 1.25 hours

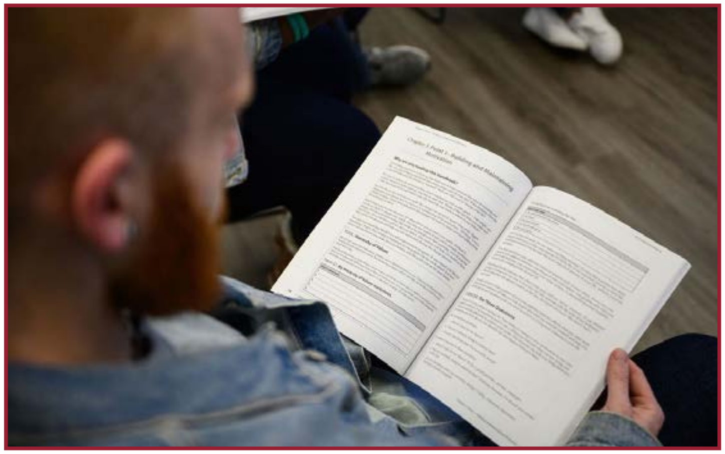

This training explains the key principles and processes of MI with examples of each, reviews the stages of change and recognizing participant language, and use of specific MI techniques such as open-ended questions, affirmations, reflective statements, and summaries (OARS), ruler/sliding scales, eliciting change talk, and reviewing sample MI skills.

Questions & Scenarios — Motivational Interviewing Workshop

MI Part 2: Q&A/Workshop

Self-paced, online; approximately 1.25 hours

This training gives RCs the opportunity to participate in a live, hands-on MI question and answer session and practice several MI techniques discussed in the prior didactic session.

The MI trainings have taught me how to do open-ended questions. When you have a participant in front of you, using the tools [from the training] helps you keep the conversation going ... and broadens the entire conversation to help participants make bigger plans than the one goal they had originally set.

HCS-VOH KY Trained RC

Harm Reduction in Healthcare and Beyond

Harm Reduction in Healthcare and Beyond

Self-paced, online; approximately 1 hour

The goal of this training is to understand the history and philosophy of harm reduction as related to drug use, articulate benefits and challenges of harm reduction and preventative medicine practices in formal healthcare settings, and employ specific harm reduction interventions for routes of administration and substances related to opioid use disorder.

Domain 4

- VOH-HCS Linkage & Retention RC Programs Overview

- HCS-VOH Data Collection Process for Dissemination

Forms:

- Enrollment Questionnaire
- Linkage Questionnaire
- Retention Questionnaire
- Discharge Questionnaire

SOP Reviews

The following set of SOPs are designed to further explain the HCS linkage and retention programs in detail. Please note that in addition to the SOPs provided below, our program has additional protocols for communication across various teams as well as protocols for implementing our barrier relief, transportation assistance, and housing assistance programs utilizing HCS grant funds. Because the programs are specific to our organization and HCS, the SOPs are not included in this manual but can be provided upon request.

Linkage and Retention SOP

Self-paced, online; approximately 15 minutes

This document describes the programs, eligibility questions, the linkage/retention process, and format and frequency for the RC recovery support sessions for the linkage and retention programs.

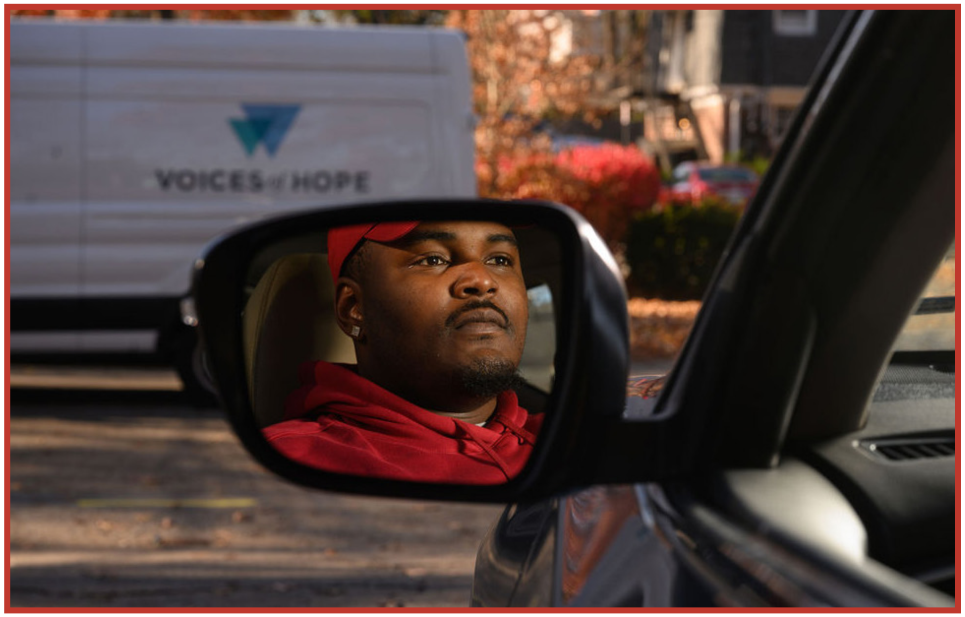

Data SOP

Self-paced, online; approximately 30 minutes

Data collection is an essential part of the HCS linkage and retention programs. The information allows the ability to track outcomes as well as easily identify and summarize key information regarding each participant's recovery plan, needed resources, etc. HCS-specific data collection training for RCs consists of a basic overview of the program, required forms, and the data collection process. The documents include the list of information documented for the linkage and retention programs as well as the SOP used to collect this data through the Research Electronic Data Capture (REDCap) system.

Domain 5

Overdose Education and Naloxone Distribution (OEND)

As part of the HCS process, it is important that all participants are educated on opioid overdose and have access to naloxone.

The following information includes videos and brochures that RCs can use to educate their participants on opioid overdose and naloxone administration. Please note that our team also has internal trainings and standard operating procedures regarding OEND processes and OEND data collection that are available upon request.

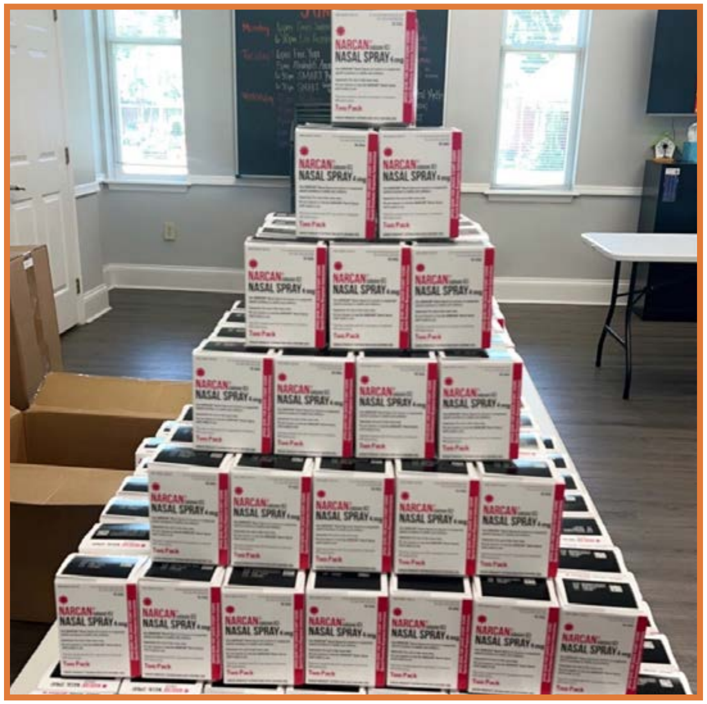

"How to Use Naloxone" Video

- English
- Spanish
- Arabic

Overdose Education Video and Training

Self-paced, online; approximately 10 minutes

This training video is available for RCs to educate participants about opioid overdose and naloxone use. We recommend RCs acquaint themselves with the information in this video to be able to talk with and educate participants about OEND. They may also share the video with participants as needed.

HCS OEND Tri-fold Brochure

- English
- Spanish
- Arabic

Overdose Education Tri-Fold Brochure

Self-paced, online; approximately 10 minutes

This brochure is another means of providing opioid overdose education that also describes how to use naloxone. We recommend RCs become familiar with the information in the brochure and provide additional education to participants using the brochure as needed.

Domain 6

Ongoing Education

KY-OPEN Recorded Sessions

We recommend that RCs continue to engage in training/educational opportunities regarding topics related to OUD. HCS offers free recordings of KY-OPEN sessions on a variety of OUD-related topics that also include CEUs. New material will continue to be added throughout 2023, and materials will be available to view for three years from their initial recording date (available until 2024–2026).

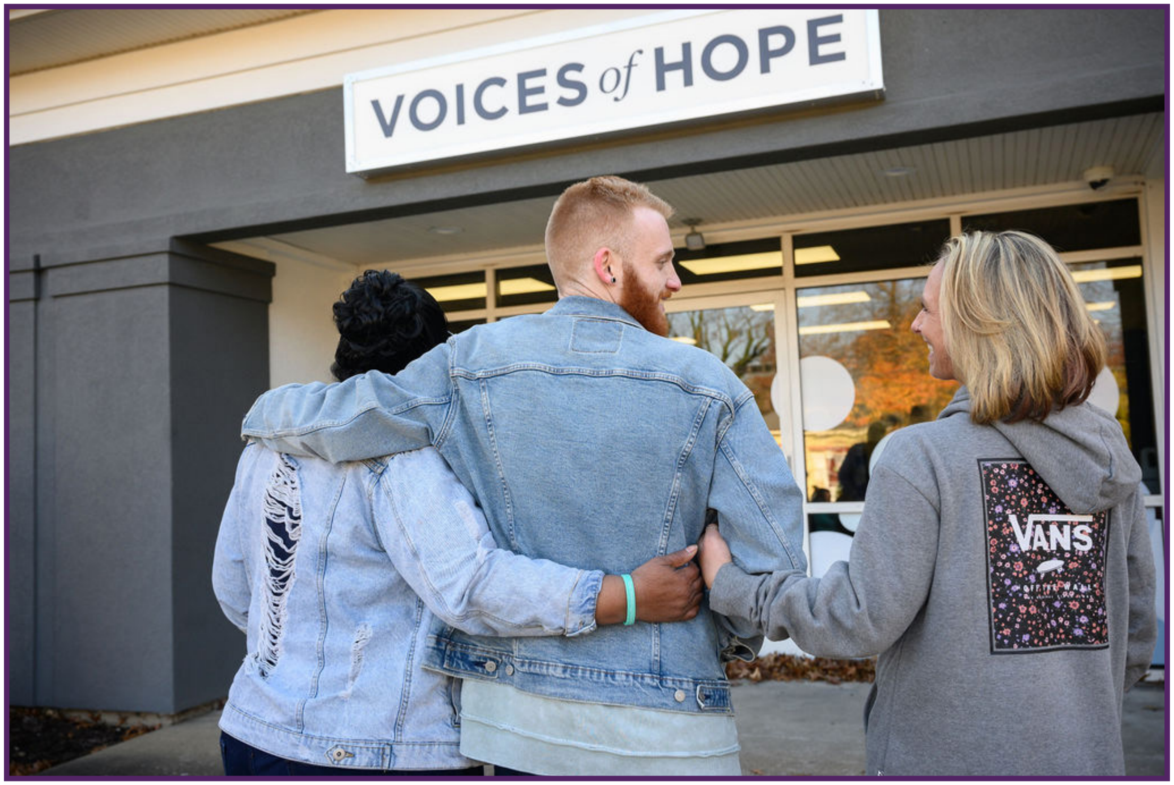

References

Ahmad, F. B., Cisewski, J. A., Rossen, L. M., & Sutton, P. (2023). *Provisional drug overdose death counts*. National Center for Health Statistics. https://www.cdc.gov/nchs/nvss/vsrr/drug-overdose-data.htm

Bardwell, G., Kerr, T., Boyd, J., & McNeil, R. (2018). Characterizing peer roles in an overdose crisis: Preferences for peer workers in overdose response programs in emergency shelters. *Drug and Alcohol Dependence, 190*, 6-8. https://doi.org/https://doi.org/10.1016/j.drugalcdep.2018.05.023

Dahlem, C. H. G., Scalera, M., Anderson, G., Tasker, M., Ploutz-Snyder, R., McCabe, S. E., & Boyd, C. J. (2021). Recovery opioid overdose team (ROOT) pilot program evaluation: A community-wide post-overdose response strategy. *Subst Abus, 42*(4), 423-427. https://doi.org/10.1080/08897077.2020.1847239

Gormley, M. A., Pericot-Valverde, I., Diaz, L., Coleman, A., Lancaster, J., Ortiz, E., Moschella, P., Heo, M., & Litwin, A. H. (2021). Effectiveness of peer recovery support services on stages of the opioid use disorder treatment cascade: A systematic review. *Drug and Alcohol Dependence, 229*, 109123. https://doi.org/https://doi.org/10.1016/j.drugalcdep.2021.109123

Jack, H. E., Oller, D., Kelly, J., Magidson, J. F., & Wakeman, S. E. (2018). Addressing substance use disorder in primary care: The role, integration, and impact of recovery coaches. *Substance Abuse, 39*(3), 307-314. https://doi.org/10.1080/08897077.2017.1389802

Krawczyk, N., Rivera, B. D., Jent, V., Keyes, K. M., Jones, C. M., & Cerdá, M. (2022). Has the treatment gap for opioid use disorder narrowed in the U.S.?: A yearly assessment from 2010 to 2019". *International Journal of Drug Policy, 110*, 103786. https://doi.org/https://doi.org/10.1016/j.drugpo.2022.103786

Larochelle, M. R., Bernson, D., Land, T., Stopka, T. J., Wang, N., Xuan, Z., Bagley, S. M., Liebschutz, J. M., & Walley, A. Y. (2018). Medication for Opioid Use Disorder After Nonfatal Opioid Overdose and Association With Mortality. *Annals of Internal Medicine, 169*(3), 137. https://doi.org/10.7326/m17-3107

Magidson, J. F., Regan, S., Powell, E., Jack, H. E., Herman, G. E., Zaro, C., Kane, M. T., & Wakeman, S. E. (2021). Peer recovery coaches in general medical settings: Changes in utilization, treatment engagement, and opioid use. *Journal of Substance Abuse Treatment, 122*, 108248. https://doi.org/https://doi.org/10.1016/j.jsat.2020.108248

National Academies of Sciences, Engineering, and Medicine; Health and Medicine Division; Board on Health Sciences Policy; Committee on Medication-Assisted Treatment for Opioid Use Disorder, Mancher, M., & Leshner, A. I. (Eds.). (2019). *Medications for Opioid Use Disorder Save Lives*. National Academies Press (US).

Wakeman, S. E., Larochelle, M. R., Ameli, O., Chaisson, C. E., McPheeters, J. T., Crown, W. H., Azocar, F., & Sanghavi, D. M. (2020). Comparative Effectiveness of Different Treatment Pathways for Opioid Use Disorder. *JAMA Network Open, 3*(2), e1920622. https://doi.org/10.1001/jamanetworkopen.2019.20622

Walsh, S. L., El-Bassel, N., Jackson, R. D., Samet, J. H., Aggarwal, M., Aldridge, A. P., Baker, T., Barbosa, C., Barocas, J. A., Battaglia, T. A., Beers, D., Bernson, D., Bowers-Sword, R., Bridden, C., Brown, J. L., Bush, H. M., Bush, J. L., Button, A., Campbell, A. N. C., . . . Chandler, R. K. (2020). The HEALing (Helping to End Addiction Long-term SM) Communities Study: Protocol for a cluster randomized trial at the community level to reduce opioid overdose deaths through implementation of an integrated set of evidence-based practices. *Drug and Alcohol Dependence, 217*, 108335. https://doi.org/https://doi.org/10.1016/j.drugalcdep.2020.108335

Winhusen, T., Wilder, C., Kropp, F., Theobald, J., Lyons, M. S., & Lewis, D. (2020). A brief telephone-delivered peer intervention to encourage enrollment in medication for opioid use disorder in individuals surviving an opioid overdose: Results from a randomized pilot trial. *Drug Alcohol Depend, 216*, 108270. https://doi.org/10.1016/j.drugalcdep.2020.108270

October 2023

VOICES of HOPE

NIH HEAL INITIATIVE

HEALing Communities Study Kentucky
