# Supplementary material for: The development of a recovery coaching training curriculum to facilitate linkage to and increase retention on medications for opioid use disorder
Source: Front Public Health. 2024 Feb 15;12:1334850. doi: 10.3389/fpubh.2024.1334850 (PMC10903364; doi:10.3389/fpubh.2024.1334850)
Supplement: Supplementary file 3 [file Image_1.PDF]

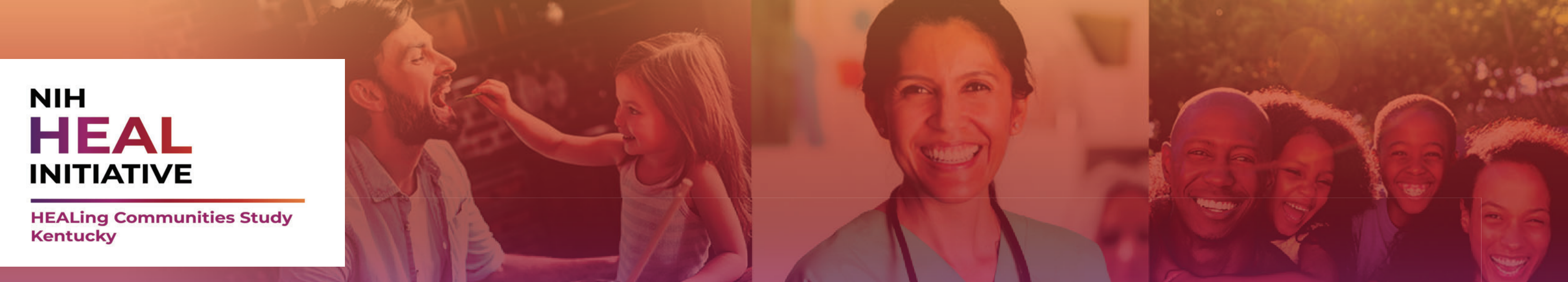

Medications for Opioid Use Disorder

Why do we use medications to treat opioid use disorder?

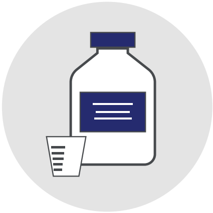

Methadone

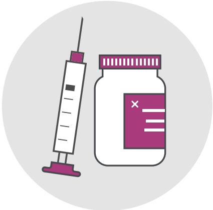

Buprenorphine (Suboxone, Sublocade, Brixadi)

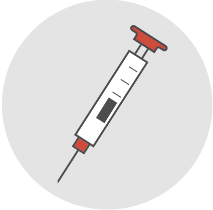

Naltrexone (Vivitrol)

Medications for opioid use disorder save lives.

People with opioid use disorder who stop using opioids often relapse. Relapse is dangerous because restarting use puts a person at high risk for a fatal overdose.

Medications lower the risk of relapse and death. They also help keep people in treatment and decrease illegal opioid use and property crime. In other words, taking medication helps people enter **remission** and stay in **recovery**.

Remission means stopping opioid use and having no symptoms of opioid use disorder.

Recovery is a process of change through which individuals improve their health and wellness, live self-directed lives, and strive to reach their full potential.

How long do you take medication for opioid use disorder?

Opioid use disorder is often a chronic illness like diabetes or heart disease. It can require treatment over many years or even for life. Treatment continues as long as the medication is helping.

Stopping medication quickly can increase the risk of overdose and death. Patients should discuss stopping with their health care provider and never stop on their own.

What drugs are opioids?

Opioids include pain pills such as oxycodone, hydrocodone, and morphine. Heroin is also an opioid. Fentanyl is a strong prescription opioid. It is sometimes made illegally and mixed into heroin or other street drugs.

How do medications for opioid use disorder work?

Opioids work at specific receptors in the brain. Think of a plug fitting into an outlet. People with opioid use disorder often feel sick and have strong cravings when no opioid is plugged in.

Methadone and **buprenorphine** plug into the receptor in the brain. They treat withdrawal, cravings , and pain.

Methadone is like a regular plug that fully activates the receptor. Buprenorphine is like a plug in a power strip that partially activates the receptor.

Naltrexone is like a plug cover that blocks the receptor without activating it. Naltrexone can't be used for about 7 to 10 days after opioid use. Overdose risk is high during that time.

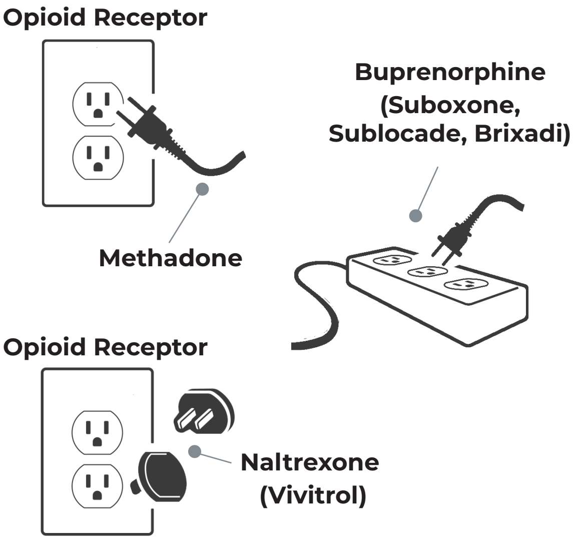

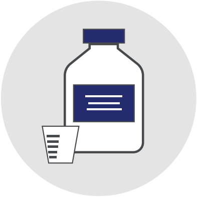

Methadone

Methadone reduces withdrawal symptoms and cravings. It can also be used to treat pain.

Methadone can reduce the effects of other opioids, which protects a person from overdose. For opioid use disorder, methadone is taken as a liquid.

Where do you get methadone?

Methadone is provided at licensed opioid treatment programs. These clinics are highly regulated and monitored by the government. People on methadone work hard to be in treatment. For the first 90 days, they may be asked to go to the clinic every day. Clinic staff watch them take each dose. Patients with opioid use disorder always get their methadone from a clinic. They do not get a prescription for methadone to fill at a pharmacy.

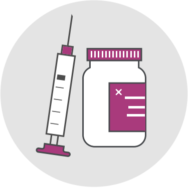

Buprenorphine (Suboxone, Sublocade, and Brixadi)

Buprenorphine reduces withdrawal symptoms and cravings. It can also be used to treat pain. Buprenorphine can reduce the effects of other opioids, which protects a person from overdose.

Most of the time, buprenorphine is taken as a film or tablet that melts under the tongue. These doses are usually taken once each day. It is also available in other forms, such as a shot that lasts one month.

Where do you get buprenorphine?

Buprenorphine is provided in a variety of settings like a doctor’s office or hospital. It can be prescribed only by health care providers. Patients fill their prescription for tablets or films at a pharmacy. Patients must visit a health care provider to receive the shot.

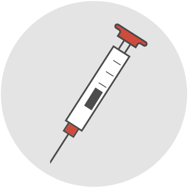

Naltrexone (Vivitrol)

Naltrexone blocks the effects of opioids. However, patients must not use opioids for about 7 to 10 days before starting naltrexone. This delay makes starting treatment difficult. Naltrexone comes as pills or a monthly shot. The shot has much better results, so it is recommended more than the pills.

Where do you get naltrexone? Naltrexone is provided in a variety of settings like a doctor's office, hospital, pharmacy, and even jails or prisons. Any prescriber can write the prescription. The shot is administered once a month by a health care provider.

	Methadone	Buprenorphine	Naltrexone
Treats Withdrawal	✓	✓	x
Decreases Cravings	✓	✓	may reduce cravings somewhat
Treats Pain	✓	✓	x
Reduces Infections	✓	✓	data not available
Reduces Crime	✓	✓	data not available
Withdrawal Needed	x	x	✓
Saves Lives	✓	✓	data not available
