# Supplementary material for: The development of a recovery coaching training curriculum to facilitate linkage to and increase retention on medications for opioid use disorder
Source: Front Public Health. 2024 Feb 15;12:1334850. doi: 10.3389/fpubh.2024.1334850 (PMC10903364; doi:10.3389/fpubh.2024.1334850)
Supplement: Supplementary file 4 [file Image_2.pdf]

Medication for Opioid Use Disorder (MOUD) Checklist Grading Rubric

Recovery coach (RC) must have an average score of >2.5 across all 5 criteria and no 1 on any single criterion to pass and be ready for deployment.

	Unsatisfactory (1)	Room for Improvement (2)	Good (3)	Excellent (4)	Score
Criteria 1 (Preparation)	RC was not in the waiting room on time AND did not have materials needed.	RC was not in waiting room on time OR did not have materials needed.	RC was on time, had materials, but needed a short time to prepare for checklist.	RC was in waiting room on time, had all materials needed, and ready to start.	
Criteria 2 (4 skills):<br>1. Knows key terms/definitions<br>2. Explains MOUD mechanism of action (MOA) using figure<br>3. Explains specifics of each medication<br>4. Explains medication effects using table	RC successfully demonstrates 0 or 1 skill OR cannot explain MOUD table.	RC successfully demonstrates 2 skills.	RC successfully demonstrates 3 skills.	RC successfully demonstrates all 4 skills.	
Criteria 3 (Case Studies and MI skills using OARS)	RC is unable to perform case study or does not know how to incorporate OARS and MI into responses.	RC can perform case study with guidance and/or uses only 1 of the OARS in responses.	RC performs case study very well and uses at least 2 of the OARS.	RC performs case study while effectively incorporating all of the OARS.	
Criteria 4 (4 skills):<br>1. Provides overdose education<br>2. Provides or informs where to get naloxone<br>3. Provides education on safe injection practices and other harm reduction material<br>4. Informs where to find local SSP	RC successfully demonstrates 0 or 1 skill OR fails to provide or inform where to get naloxone.	RC successfully demonstrates 2 skills.	RC successfully demonstrates 3 skills.	RC successfully demonstrates all 4 skills.	
Criteria 5 (Linkage & Retention Programs – 2 skills):<br>1. Explains purposes of each program<br>2. Knows frequency (freq.) of contact in each program and how each freq. is determined	RC has no knowledge of Linkage & Retention Programs.	RC explains purpose of Linkage & Retention Programs but cannot explain freq. of contact and how freq. is determined.	RC explains purpose of Linkage & Retention Programs, freq. of contact, and how freq. is determined with some guidance.	RC explains purpose of Linkage & Retention Programs, freq. of contact, and how freq. is determined without any guidance.	

Total Score/5= Average Score ___________

Result: Pass ☐ Recommend Retest ☐

8/1/2023, TT & VOH
